# Refined preferences of prioritizers improve intelligent diagnosis for Mendelian diseases

**DOI:** 10.1038/s41598-024-53461-x

**Published:** 2024-02-03

**Authors:** Xiao Yuan, Jieqiong Su, Jing Wang, Bing Dai, Yanfang Sun, Keke Zhang, Yinghua Li, Jun Chuan, Chunyan Tang, Yan Yu, Qiang Gong

**Affiliations:** 1 Changsha Kingmed Center for Clinical Laboratory, Lutian Road 28, Changsha, 410000 Hunan China; 2 Guangzhou Kingmed Center for Clinical Laboratory, Guangzhou, Guangdong China; 3 Genetalks Biotech. Co., Ltd., Changsha, Hunan China

**Keywords:** Software, Genetic testing, Bioinformatics, Molecular medicine

## Abstract

Phenotype-guided gene prioritizers have proved a highly efficient approach to identifying causal genes for Mendelian diseases. In our previous study, we preliminarily evaluated the performance of ten prioritizers. However, all the selected software was run based on default settings and singleton mode. With a large-scale family dataset from Deciphering Developmental Disorders (DDD) project (N = 305) and an in-house trio cohort (N = 152), the four optimal performers in our prior study including Exomiser, PhenIX, AMELIE, and LIRCIAL were further assessed through parameter optimization and/or the utilization of trio mode. The in-depth assessment revealed high diagnostic yields of the four prioritizers with refined preferences, each alone or together: (1) 83.3–91.8% of the causal genes were presented among the first ten candidates in the final ranking lists of the four tools; (2) Over 97.7% of the causal genes were successfully captured within the top 50 by either of the four software. Exomiser did best in directly hitting the target (ranking the causal gene at the very top) while LIRICAL displayed a predominant overall detection capability. Besides, cases affected by low-penetrance and high-frequency pathogenic variants were found misjudged during the automated prioritization process. The discovery of the limitations shed light on the specific directions of future enhancement for causal-gene ranking tools.

## Introduction

The successful deciphering of the human genome sequence at the beginning of this century and the follow-up rise of massively parallel sequencing technology changes the paradigm of research and diagnosis for genetic disorders. Massively parallel sequencing, also called high-throughput sequencing or next-generation sequencing (NGS), produces millions to billions of short reads (generally 30 to 300 bases long) per instrument run, making determining individual genome or exome at affordable cost possible. After the first use of whole-exome sequencing (WES) to seek the causal gene in a patient with Miller syndrome in 2010^1^, NGS technology is becoming part of routine clinical practice in the diagnosis of monogenic genetic diseases^2,3^, otherwise called Mendelian diseases. For some of this kind of disease, patients stratified into groups each with a common diagnostic molecular profile had benefited from a tailored treatment, management, or prevention strategy^4^, demonstrating the potential power of precision medicine. In order to comprehensively elucidate the associations between genetic variants and human phenotypes to further set the stage for personalized therapy in public healthcare, several countries launched population-wide genome sequencing projects^5–9^. In one of the earliest projects on rare disease, Deciphering Developmental Disorders (DDD) project, over 4500 children patients have been diagnosed via WES, and dozens of novel genes for developmental disorders have been confirmed^10,11^. Children with developmental disorders across the world also get the chance to be diagnosed through information shared in DECIPHER^12^. Besides, some diagnosed children with specific causal variants obtained opportunities to participate in treatment trials.

Manually pinpointing the disease-causing gene/variant in the patient genome/exome sequencing result has always been tricky^13^. Emerging causal-gene prioritizers utilize the accurate phenotyping results of patients, an internal sophisticatedly-constructed genotype–phenotype knowledge repository, and variant characteristics such as allele frequency (AF) and predicted pathogenicity, to prioritize candidate genes by calculated probability. This kind of advanced computational tool has proved a highly efficient approach to assist in identifying the “culprit” for inherited diseases^14,15^, and one of the recognized prioritizers, Exomiser^16^ had been implemented in the diagnostic pipeline of a pilot study in the U.K. 100,000 Genomes Project (UK100K)^17^. In our previous study^18^, the performance of ten phenotype-guided disease-causing gene prioritizers was benchmarked using two well-curated datasets, and all software was run based on default settings and singleton mode. This very first step of our scheduled research series identified LIRICAL^19^ and AMELIE^20^ as outstanding competitors. Generally, parameter tuning is necessary for software to achieve optimal operational status, and the extra parental genetic information could facilitate the causal-gene identification upon the proband variant analysis. Thus, herein, as the second step of our entire research project, further analysis was conducted to assess some of the best-performing prioritizers in our prior study through parameter optimization and/or the utilization of trio mode. In this continuative study, a total of 457 family datasets were used for assessment of phenotype-guided causal-gene prioritization software. Major parameters of each software were investigated and the impacts elicited by these parameters were quantified and visualized. In addition, misjudgments and missed diagnoses were fully troubleshot which revealed limitations of assessed prioritizers. We expect the presented work could provide valuable information for the field of computer-assisted rapid diagnosis, namely intelligent diagnosis in Mendelian diseases. We also believe the community could benefit from our series of evaluative research.

## Methods

### DDD and KGD trio dataset

For this assessment study, phenotypic data represented by Human Phenotype Ontology (HPO) terms and genotypic data recorded as Variant Call Format (VCF) files of 305 positive-diagnosed patients with developmental disorders in the DDD project were utilized. This proband cohort was the same as used in our previous study^18^. Additionally, the VCF files and the disease status information of the parents of the patient cohort were downloaded from European Genome-Phenome Archive^21^ with authorization, and then integrated into the proband cohort data, generating a final 305-trio dataset. Each proband carried a single causal gene identified through an elaborate variant filtering pipeline^22^. Specifically, variants were filtered by orderly considering population allele frequency, predicted functional consequence, genomic location, variant type, and inheritance. After the above-automated procedures, casual variant(s) would be presented in the final report through strict manual review for relevance to the individuals’ phenotypes, and other aspects.

The in-house Kingmed Genetic Disorder (KGD) patient cohort in our previous study was updated: only 58 proband cases were kept for the presence of both genotypic data and disease status information from their parents. Another 94 eligible patient cases enrolled between 2021 and 2022, and subjected to trio-based WES were added. Finally, the updated in-house cohort contained 152 three-member families. The exonic DNA fragments of the blood samples of both probands and parents were captured using xGen™ Exome Research Panel kit and sequenced by Illumina Nextseq 550 sequencer, and the VCF files were generated and filtered following the Genome Analysis Toolkit (GATK) Best Practices Workflow for germline short variant discovery^23^. Consistent with our prior study, the variant interpretation procedure was rigorously executed under the American College of Medical Genetics and Genomics/Association for Molecular Pathology (ACMG/AMP) guidelines^24^ and its refinement^25–28^. Each of the total 152 probands carried a single Sanger-confirmed pathogenic gene (Supplementary Appendix I). HPO terms were accurately extracted from the patient’s medical records and the KGD cohort consisted of cases with various congenital abnormalities (Supplementary Appendix I).

HPO terms of each patient from the DDD and KGD cohort were reviewed for validity and obsolete terms were updated. An HPO term was assigned to its parent class by using the ‘ontologyIndex’ package in R software (version 4.0).

### Prioritizers assessed in this study

At the time of this writing, the latest stable version of each of the four best-performing prioritizers in our prior study including Exomiser, PhenIX^29^, AMELIE and LIRICAL was selected. Of note, Xrare^30^ was excluded from this assessment due to its unavailability. The four selected prioritizers were all free for academic use, and took HPO terms and VCF files as input. The brief introductions of these prioritizers were deposited in Supplementary Sect. S1.

### Visualization and statistical analysis

The cumulative distribution function (CDF) curves, bar plots, stacked bar plots, violin plots, half-violin half-dot plots, and pie plots presented in this work were performed by using the ‘ggplot2’ package in R software (version 4.0). The CDF plots illustrate the percentage of cases with causal genes ranked within the top k by each method. k could be any integer between 1 and 50 (inclusive). The stacked bar plots illustrate the relative proportion of each group involving cases with causal genes ranked within a designated range. The Venn diagrams were plotted using the ‘VennDiagram’ R package. The waterfall plots and co-occurrence and mutual exclusivity analysis were performed via Oncoprinter in cBioPortal^31^. To measure the sensitivity of each prioritizer under different protocols, the proportions of cases with causal genes ranked in the top-1, and within the top-5, -10, -20, -30, -40, and -50 were calculated respectively.

### Ethical approval

This study was approved by the Ethics Committee of Guangzhou Kingmed Center for Clinical Laboratory (No. 2023006). Informed consent on research participation and data publication was signed by each KGD patient or patient’s guardian. All methods were performed in accordance with the provisions of the Declaration of Helsinki.

## Results

**Figure 1:**
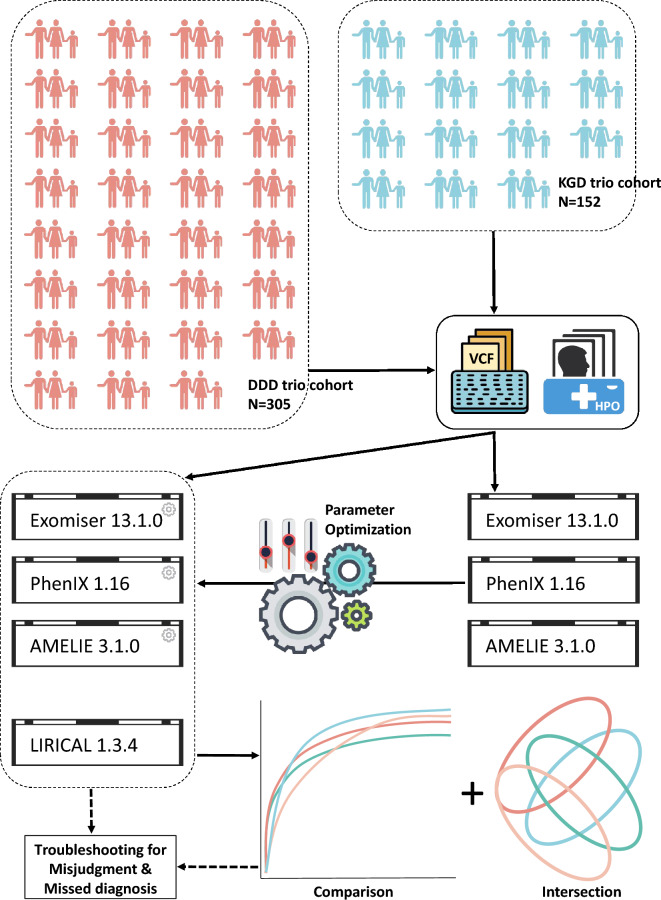
Illustration of study workflow. The VCF files and HPO terms of the DDD trio cohort (N = 305, represented as light red) and KGD trio cohort (N = 152, represented as sky blue) are well curated as input for the parameter optimization of prioritizers including Exomiser 13.1.0, PhenIX 1.16 and AMELIE 3.1.0. Then, these three ranking tools under optimal state, as well as LIRICAL 1.3.4 which have little parameter option are gathered for performance comparison using cohort data. Besides, the intersection analysis of solved cases from different prioritizers is conducted. Finally, misjudgment and missed diagnosis in the study are summed up and troubleshot.

The DDD trio dataset (N = 305) and the in-house KGD trio dataset (N = 152) were employed for this prioritizer assessment (Fig. 1). The average amount of the proband Human Phenotype Ontology (HPO) term for the two cohorts were 7.5 and 3.1, respectively, while the average amounts of the variants in the proband Variant Call Format (VCF) files were 100,033 and 108,035. Other characteristics of the DDD and KGD trio cohort such as the sex and age distribution, the amount of the unique causal genes, and the frequency of recurring causal genes and HPO terms, were described in Supplementary Sect. S2.

### Performance improvement assessment for prioritizers based on major parameter optimization

Parameter options were generally provided by the software to satisfy users’ own needs for actual situations. Optimizations in key parameters of prioritizers involved in this study were conducted first.

**Figure 2:**
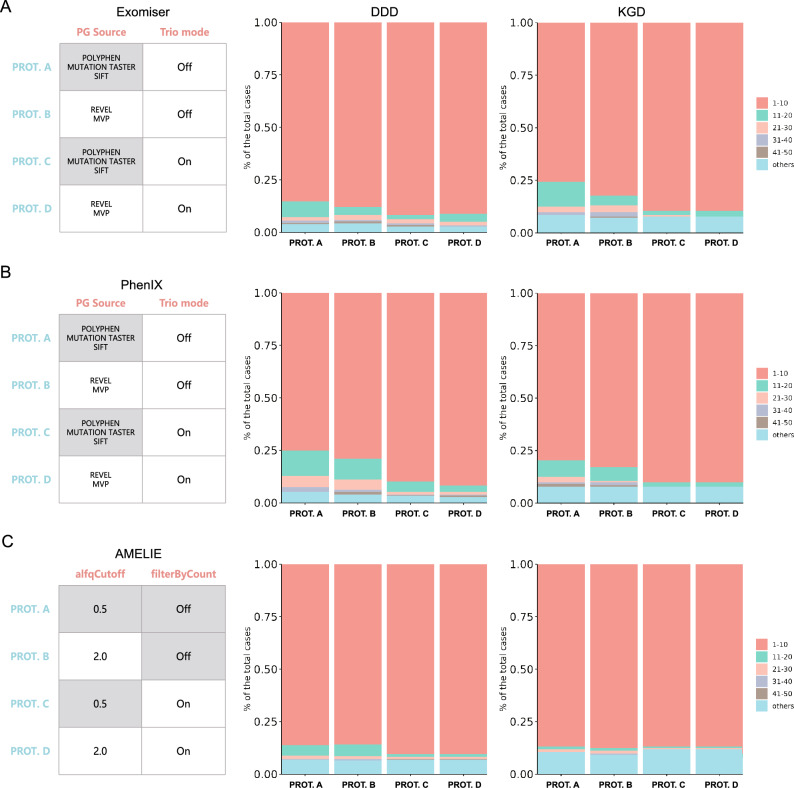
Performance improvement by parameter optimization for Exomiser, PhenIX, and AMELIE. The illustration of performance improvement by parameter optimization for Exomiser (**A**), PhenIX (**B**), and AMELIE (**C**). The tables on the left display parameter (column) options or states under different protocols (row). Grey background indicates that this option or state is the default one for a prioritizer. PG Source: pathogenicity source. The stacked bar plots illustrate the relative proportion of each group involved cases with causal genes ranked within a designated range (e.g. 1–10) for DDD (middle) and KGD (right) cohorts. Each group is represented by a different color. The labels under X-axis correspond to the protocol names in the tables on the left.

Exomiser utilized PolyPhen^32^, MutationTaster^33^, and SIFT^34^ as the “pathogenicity sources” by default for variant pathogenicity prediction in old versions including version 12.1.0 used in our previous study^18^. In the latest version 13.1.0, REVEL^35^ and MVP^36^ replace the three classic tools in the preset configuration files. Thus, the impact of pathogenicity sources on the ranking performance of Exomiser was worth exploring. Using REVEL and MVP as pathogenicity sources (Fig. 2A Protocol B) raised the proportions of cases with causal genes ranked within the top 10 of the ranking lists (referred to as “group 1–10”, represented as light red) in both cohorts. Compared with the “old” default setting (Fig. 2A Protocol A), however, the overall detection rate (defined as the proportion of cases with causal genes ranked within the top 50) was not improved noticeably in the KGD dataset (91.4% to 92.8%, Table 1) and even slightly decreased in the DDD dataset (96.1% to 95.7%). Besides, in both datasets, the case amounts of group 1–10 elevated under trio mode (Fig. 2A Protocol C and D) in comparison to singleton mode (Fig. 2A Protocol A and B). However, for KGD dataset, the overall detection rates under Protocol C and D were decreased by 0.7% compare to Protocol B (Fig. 2A, Table 1). Upon closer examination, Exomiser in trio mode failed to identify the causal genes of two KGD cases, NP22F4236 and NP22FW1164 due to the disease incomplete penetrance (Supplementary Sect. S3, Table 2).

**Table 1:** Sensitivity of each prioritizer under different protocols in each top-level experiment.

	Prioritizer	PROT	Top1 (%)	Top5 (%)	Top10 (%)	Top20 (%)	Top30 (%)	Top40 (%)	Top50 (%)
DDD	Exomiser	A	31.8	73.1	85.2	92.8	94.4	95.4	96.1
		B	53.1	83.3	87.9	91.8	94.1	94.8	95.7
		C	48.5	85.6	91.8	93.8	95.7	96.4	97.4
		D	61.6	86.6	91.1	95.1	96.7	97.4	97.4
	PhenIX	A	31.1	60	75.1	87.2	92.5	94.8	94.8
		B	37	67.9	79	88.9	93.8	94.8	96.1
		C	46.2	77.7	89.8	94.8	96.1	96.4	96.7
		D	49.5	80	91.8	94.8	96.1	96.4	97.4
	AMELIE	A	46.6	81.3	86.2	91.1	92.8	93.1	93.1
		B	46.6	81.3	85.9	91.5	92.8	93.4	93.4
		C	50.5	83.3	90.5	91.8	92.8	92.8	93.1
		D	50.5	83	90.5	91.8	92.8	92.8	93.1
	LIRICAL	–	46.6	75.4	83.3	91.8	93.8	95.7	95.7
KGD	Exomiser	A	30.3	65.1	75.7	87.5	90.1	91.4	91.4
		B	48	76.3	82.2	86.8	90.1	92.1	92.8
		C	63.2	88.2	89.5	91.4	92.1	92.1	92.1
		D	68.4	85.5	89.5	92.1	92.1	92.1	92.1
	PhenIX	A	38.8	70.4	79.6	87.5	90.1	90.8	92.1
		B	47.4	75	82.9	89.5	90.1	91.4	92.1
		C	65.8	86.2	90.1	92.1	92.1	92.1	92.1
		D	69.1	86.2	90.1	92.1	92.1	92.1	92.1
	AMELIE	A	50.7	79.6	86.8	88.2	89.5	89.5	89.5
		B	52	80.3	87.5	88.8	90.1	90.8	90.8
		C	54.6	81.6	86.8	87.5	88.2	88.2	88.2
		D	55.9	81.6	86.8	87.5	88.2	88.2	88.2
	LIRICAL	–	58.6	75.7	85.5	94.1	95.4	96.1	96.1

**Table 2:** Information of causal variants of misjudged cases and missed diagnoses.

Type	Case	Gene	Variant (hg19)	cDNA AA change	HGMD ClinVar	Count max AF	Zygosity origin	Note
Misjudged case	NP22F4236	PRRT2	16-29825015-G-GC	c.641dupC p.Arg217Profs*8	DM P/LP	514/0 0.47%	Het Maternal	Healthy parental carrier GnomAD allele count > 3
	NP22FW1164	DEPDC5	22-32211195-C-T	c.1663C>T p.Arg555Ter	DM P	1/0 0.006%	Het Paternal	Healthy parental carrier
	DDDP110879	GJB2	13-20763685-AC-A	c.35delG p.Gly12Valfs*2	DM P	1737/10 0.96%	Hom Biparental	Max AF > 0.5%
	NP23FW3882	PRRT2	16-29825015-GC-G	c.641delC p.Arg217Glufs*12	DM P/LP	718/0 0.96%	Het Maternal	
	NP24FW2307	G6PD	X-153760472-C-T	c.1388G>A p.Arg463His	DM P	106/1/33 0.70%	Het Maternal	
	NP23FW1402	PRRT2	16-29825015-G-GC	c.641dupC p.Arg217Profs*8	DM P/LP	514/0 0.47%	Het Paternal	GnomAD allele count > 3
Missed diagnose	DDDP102392	ARID1A	1-27105553-C-T	c.5164C>T p.Arg1722Ter	DM P	NA	Het De novo	Non-”PASS” tag in the VCF FILTER column
	DDDP111661	ARID1A	1-27106354-C-T	c.5965C>T p.Arg1989Ter	DM P	NA	Het De novo	
	DDDP110127	KANSL1	17-44248419-G-T	c.1091C>A p.Ser364Ter	DM NA	NA	Het De novo	
	DDDP102205	TUBA1A	12-49580580-C-T	c.40G>A p.Val14Ile	DM NA	NA	Het De novo	
	DDDP112654	KCNH1	1-211093309-G-C	c.1135C>G p.Leu379Val	DM P	NA	Het De novo	
	DDDP112720	CDK13	7-40039066-G-A	c.2149G>A p.Gly717Arg	DM P	NA	Het De novo	
	DDDP111060	LRP2	2-170068598-C-T	c.6160G>A p.Asp2054Asn	DM? Conflicting	275/0 0.25%	Comp. Het Maternal	
			2-170103472-G-A	c.2933C>T p.Thr978Met	DM? Benign	1078/8 2.2%	Comp. Het Paternal	
	NP22F3138	CYP21A2	6-32006858-C-G	c.293-13C>G	DM P	557/0 0.37%	Hom Biparental	

*DM* disease-causing mutation, *DM?* likely disease-causing mutation, *P* pathogenic, *LP* likely pathogenic, *Max AF* max population allele frequency in gnomAD, *Het.* heterozygous, *Hom.* homozygous, *Comp. Het.* compound heterozygous, *In the Count & Max AF column, the value of Count follows this format* heterozygote count/homozygote count/hemizygote count (recorded in gnomAD).

As mentioned in the Supplementary Sect. S1, PhenIX shared a partial internal framework with Exomiser. Therefore, identical major parameter protocols were applied to assess PhenIX. The results showed that groups 1–10 in both datasets expanded after changing the default pathogenicity sources to REVEL and MVP (Fig. 2B Protocol B), which was similar to the situation of Exomiser. The proportions of cases with causal genes identified within the top 50 increased by 1.3% in the DDD dataset and were unchanged in the KGD dataset (Table 1). Converted to trio mode (Fig. 2B Protocol C and D), 89.8–91.8% of cases (Table 1) could be diagnosed by reviewing no more than ten candidate genes in the prioritization lists.

By changing the default pathogenicity sources to REVEL and MVP, more causal genes broke into the top 10 of the ranking results of Exomiser and PhenIX, which facilitate efficient target locking for users. Besides, consistent with expectations, the addition of phenotypic and genotypic information from parents could narrow down the outcome gene lists and thereby further boost prioritization capability. After comprehensive consideration, Protocol D was chosen as the optimal solution for both Exomiser and PhenIX in this study.

In the parameter testing of AMELIE, AF cutoff (alfqCutoff, default = 0.5%) was set to 2.0% in the first place. Loosening this parameter slightly impaired the software performance in the top-10 experiment in the DDD dataset while mildly improving that of the KGD dataset (Fig. 2C Protocol B, Table 1). Moreover, the overall detection rates in the two datasets increased by 0.3% and 1.3% respectively compared to Protocol A (Table 1). To be exact, three cases missed in the default cutoff (0.5%) were detected under a looser AF threshold (2.0%). Each of the lost-and-found cases was affected by a pathogenic variant with relatively high AF (> 0.5%) in at least one subpopulation in gnomAD^37^. These cases included DDDP110879 from the DDD dataset, and NP23FW3882 and NP24FW2307 from the KGD dataset (Supplementary Sect. S3, Table 2).

Next, the “filterByCount” option was investigated for AMELIE. This option was turned off by default and if enabled, all variants with a gnomAD homozygous count greater than or equal to a specified threshold (1 by default) would be removed, and then all remaining single heterozygous variants would be further filtered by a designated gnomAD allele count cutoff (3 by default). In the DDD dataset, switching on filterByCount significantly increased the proportion of cases with causal genes ranked within the top 10 but did not boost the overall detection rates (Fig. 2C Protocol C and D, Table 1). Notably, enabling the same option mildly shrank the total proportion of cases whose disease-causing genes were prioritized among top 50 (represented in multiple colors except sky blue) with (Protocol D) and without (Protocol C) alfqCutoff setting to 2.0 in the KGD dataset (Fig. 2C, Table 1). On further inspection, two KGD cases, NP22F4236 and NP23FW1402 were filtered by AMELIE for the excessive gnomAD allele count (Table 2). The former was also misdiagnosed by trio-mode Exomiser due to the healthy parental carrier while the latter was not because the father also suffered from a seizure.

As shown in Fig. 2C and Table 1, Changing alfqCutoff to 2.0 and turning off filterByCount could reduce the misjudgments mentioned above to improve the overall detection rate of AMELIE. Hence, Protocol B was relatively appropriate for this online tool in our study.

### Performance benchmarking for optimized prioritizers across different experiments

**Figure 3:**
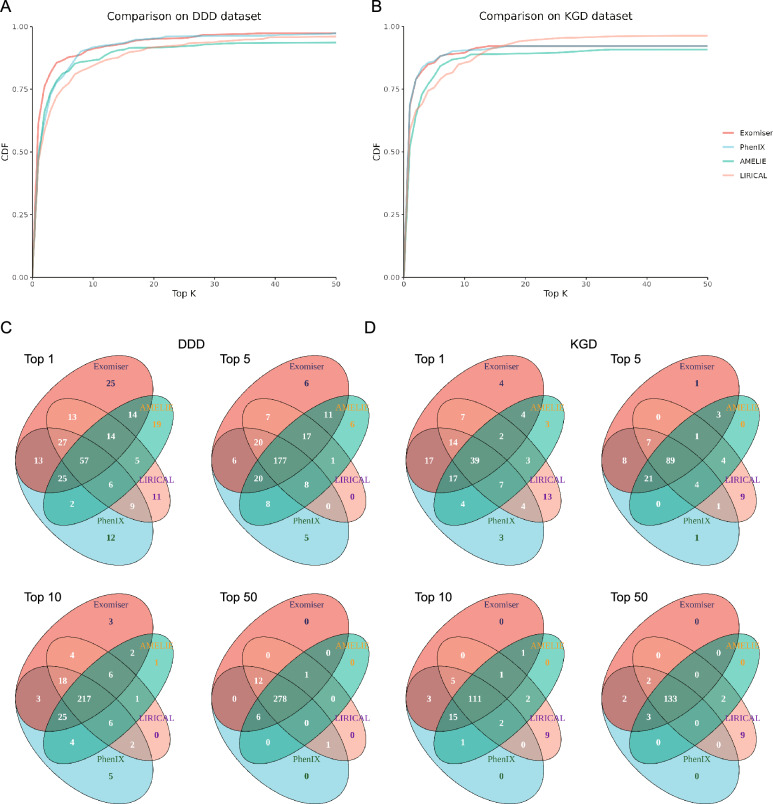
Performance benchmarking for optimized prioritizers across different experiment settings. Performance benchmarking for Exomiser under Protocol D, PhenIX under Protocol D, AMELIE under Protocol B, and LIRICAL across different experiment settings. The CDF plots (**A,B**) illustrate the percentage of the DDD and KGD cases with causal genes ranked within the top k by each prioritizer. k could be any integer between 1 and 50 (inclusive). Each prioritizer is represented by a different color. The Venn diagrams (**C,D**) illustrate the amounts of intersected solved cases of different ranking tools in top-1, -5, -10, and -50 experiment settings.

In the next step, the performance of Exomiser under Protocol D, PhenIX under Protocol D, AMELIE under Protocol B, and LIRICAL which had little parameter option, were gathered for comparison. In the DDD dataset (Fig. 3A), Exomiser outshone others in the top-1 and -5 experiments. It correctly assigned the causal gene at the very top and within the top 5 in 61.6% and 86.6% of the total 305 DDD trio cases (Table 1). PhenIX caught up to Exomiser in the top-10 experiment and these two prioritizers twisted each other in the following experiments. Finally, in the top-50 experiment, they both solved 97.4% of the total DDD cases (Table 1). In most experiment settings, Exomiser and PhenIX under Protocol D defeated Amelie and LIRICAL, the best two performers in our previous study, showing the advantage of trio mode. In the KGD dataset (Fig. 3B), Exomiser and PhenIX led the competition in the initial stage. However, LIRICAL stood out in the top-20 experiment and gradually expand the leading edge. Eventually, LIRICAL captured the causal genes within the top 50 for about 96.1% KGD cases, ahead of both Exomiser and PhenIX by about four percentage points (Table 1). What should be emphasized was that despite updating of the versions of AMELIE (from version “Oct5 2020” to 3.1.0) and LIRICAL (from version 1.3.0 to 1.3.4), as well as the genotypic and phenotypic data of the in-house cohort, the situations between AMELIE and LIRICAL in both DDD and KGD datasets were roughly followed the findings in our previous study: Amelie outperformed LIRICAL in the front part of the CDF curves, and was surpassed by LIRICAL in the latter half.

In general, the four software under their optimum conditions situated 46.6–69.1% of the causal genes at the highest positions of the ranking results (Table 1). Specifically, Exomiser put the right genes at the very top in 61.6% of the DDD cases and 68.4% of the KGD cases and hence became the only one with the capability to directly hit the target in more than 60% of cases in both datasets. Of note, 83.3–91.8% of the causal genes were placed among the very first ten candidates in the prioritization lists by the four tools (Table 1, Fig. 3A,B). Lastly, the overall detection rates ranged from 90.8 to 97.4% (Table 1, Fig. 3A,B). In this dimension, favorable stability was observed in LIRICAL: the true genes were emerging within the top 50 for more than 95% of cases across different cohorts.

The case intersections across different experiment settings were also explored. In the top-1 experiment in the DDD dataset, Exomiser, PhenIX, AMELIE, and LIRICAL successfully diagnosed 188, 151, 142 and 142 cases respectively. The amount of intersected cases of the four was 57, and prioritizer-unique cases were 25, 12, 19 and 11 respectively (Fig. 3C). In the following experiments, the intersected cases increased while the prioritizer-unique cases decreased gradually. In the final top-50 experiment, the intersected cases were up to 278 and the prioritizer-unique cases were all zeros (Fig. 3C). In summary, a total of 298 of 305 (97.7%) cases were solved by either of the four prioritizers with causal genes ranked within the top 50 of candidate lists. In the KGD dataset, the tendencies of the amount of intersected cases and prioritizer-unique cases across experiment settings (Fig. 3D) were consistence with that of the DDD dataset. In the final top-50 experiment, four prioritizers shared up to 133 solved cases. LIRICAL possessed nine unique solved cases eventually (Fig. 3D) and this made it the highest in CDF curves (Fig. 3B). To sum up, 99.3% of the causal genes (151/152) of the KGD cohort were successfully captured within the top 50 by at least one of the four software.

The target genes of seven DDD cases and one KGD case were not present in the result lists produced by either of the four prioritizers (Table 2 “Missed diagnose” Section). Specially, case DDDP111060 was affected by a compound heterozygous variant and case NP22F3138 carried a pathogenic homozygous intron variant.

### Performance alteration measurement for Exomiser along with minor factor adjustment

Exomiser provided comprehensive parameter options for users to precisely modify its behavior in the prioritization procedure. In the final step, the performance alteration elicited by the adjustment of minor parameters or factors was measured for Exomiser using the DDD dataset (Supplementary Sect. S4). The function of one of the Exomiser’s option named Failedvariantfilter (FVF) was to help to remove low-quality variants which were not flagged as PASS or “.” in the FILTER column of a VCF file. This filtering function was highly recommended by the authors of Exomiser and was switched on by default in version 13.1.0 used in this study. By dissecting the causal-gene ranking trends in the performance alteration measurement for Exomiser along with minor factor adjustment (Supplementary Appendix II), the causal genes of six unsolved DDD cases (except DDDP111060) were found excluded by Exomiser’s FVF option. Further scrutinization of the VCF files revealed that the disease-causing variants of the six were not passed certain filters set up by the DDD project so the FILTER column of these variants was not marked as PASS or “.”.

## Discussion

In this study, a detailed assessment of four causal-gene prioritizers was conducted using a total of 457 family datasets. The impacts of major software parameters and the effects produced by the adjustment of some minor parameters or factors were rigorously investigated. The improvements brought by refined preferences of prioritizers were quantified and visualized. However, some limitations and issues related to parameters should be emphasized: (1) This study only considered universal parameters for batch testing in the context of cohort-level assessment. Prioritizer performance could be further enhanced via case-level customized parameter settings by tuning certain other parameters. For example, for the missed KGD case NP22F3138 (Table 2) affected by a homozygous intron variant, if the “CODING_TRANSCRIPT_INTRON_VARIANT” option under the “variantEffectFilter” parameter section in the Exomiser configuration file is commented out (that means keeping intron variants), the causal gene will appear on the final ranking list, rather than be totally excluded by Exomiser. This case-by-case exploration with personalized parameter configuration is one of our future tasks. (2) Some parameters are strongly recommended to keep open by the tool. For example, the “onlyPassVariants” option of AMELIE is activated by default to escaping from noises brought about by low-quality variants in the VCF files. Some other parameters produce little impact on the software performance. For instance, one of the Exomiser’s parameter “outputContributingVariantsOnly”, merely influences the variant output list, rather than the gene prioritization result. Systematically investigating these two kinds of parameters is not necessary because it would provide potentially redundant information for the audiences. What needs clarification is that each parameter of the four assessed tools was deeply discussed before the start-up of this study, and to exhibit the most useful information to the audiences, the effects of limited parameters are carefully probed during the research. (3) As with the tested parameters in our study, users should weigh them circumstantially. For example, AMELIE did have a better overall detection rate with a loosened alfqCutoff and a turned-off filterByCount option according to our findings (Fig. 2C). However, the reverse setting of these two parameters theoretically has a positive effect on the exclusion of benign variants, and thus should be seriously considered in a real scenario. (4) AMELIE was assessed only in singleton mode because trio mode was unavailable at the time of this writing. Similarly, the trio mode of LIRICAL was under development according to the e-mail response from the author.

Through the dissection of six misjudgments in our study (Table 2), limitations in handling low-penetrance and high-frequency pathogenic variants during the automated prioritization process are disclosed. These peculiar variants were quite susceptible to the routine variant filtration mechanism inside of prioritizers, and should be protected by an exemption regulation. Although prioritizers such as Exomiser offered the option of allowing for incomplete penetrance by retaining variants in unaffected family members^14^, it would substantially increase the candidate variants to review per case, and thus partially offset the positive effects (narrowing down the candidate pool) brought by the trio mode in improving ranking performance. Curating of a comprehensive whitelist of reported (and even predicted) low-penetrance and high-frequency pathogenic variants, and then integrating it into the intelligent diagnosis workflow is a direct way to resolve the issues, and one of our future objectives. Eight missed diagnoses occurred in this study (Table 2) and six of them were caused by the non-”PASS” tags in the FILTER columns of the VCF files. Generally, it is quite reasonable for the prioritizers to pick only passed variants for the downstream analysis in order to escape from noises brought about by low-quality variants. Given this, users should be cautious about the stringency of their variant filtration procedure before inputting VCF to Exomiser with FVF enabled or other prioritizers with similar filtering functions.

According to the statistics in Table 1, a user only needs to review the first ten candidate genes to make a final clinic decision in more than 80% cases, with a optimized prioritizer. To pinpoint the true culprit during candidates reviewing, local population allele frequency and homozygote count of variant in the population could be used to help to remove benign variants; besides, the match degree between the principal phenotypes of a patient and the involved phenotypes of a candidate gene should be thoroughly considered; moreover, the summarization of variant-related literature especially those curated by HGMD^38^ and ClinVar^39^ is indispensable for the ACMG/AMP-based classification workflow, and the direct evidence from literature as well as the ACMG/AMP class would be important bases for judging the real causal gene.

The combination of prioritizers is a strategy to further increase diagnostic yield because some prioritizers hold different sets to others in successful cases during prioritization^18^. This potential complementarity is also observed in this study (Fig. 3C,D). Fan et al. recruited a large cohort with rare genetic diseases to assess the efficacy of three prioritizers and their ensemble^40^. The research team found that integrating these three tools by the weighted-sum entropy method outperformed any single algorithm. However, biases might exist and interfere with the analysis conclusions according to the authors’ claim.

A research team in Spain benchmarked causal-gene prioritization tools with WES data of 61 unrelated singleton cases^41^, and a comparison between their study and ours was made (Supplementary Sect. S5).

With joint endeavors of the whole community, we believe that the casual-gene prioritization software will be more robust, and the field of Mendelian disease diagnosis will be eventually revolutionized by this kind of advanced tools.

### Supplementary Information

Supplementary Information.

## Data Availability

The DDD dataset underlying this article is deposited in the European Genome-Phenome Archive (https://ega-archive.org/studies/EGAS00001000775) by the Data Access Committee for DDD Project. The VCF files in the KGD dataset will be shared only for research purposes on reasonable request to the corresponding author.
