# Supplementary material for: Refined preferences of prioritizers improve intelligent diagnosis for Mendelian diseases
Source: Sci Rep. 2024 Feb 3;14:2845. doi: 10.1038/s41598-024-53461-x (PMC10838329; doi:10.1038/s41598-024-53461-x)
Supplement: Supplementary file 1 — Supplementary Information. [file 41598_2024_53461_MOESM1_ESM.docx]

# Section S1 Brief introduction of assessed prioritizers

Exomiser^1^ was featured by the calculation of the phenotypic similarity between a submitted case and curated phenotype data of humans, mice, and zebrafish. In version 13.1.0 (<https://github.com/exomiser/Exomiser/releases>, database version: 2209), a single main result file containing ranked genes/variants for all the assessed modes of inheritance (MOI) superseded a series of MOI-specific output files. For this study, the highest rank of each known causal gene in the main result file was recorded.

PhenIX^2^ evaluated and ranked a variant based on the combination of the "variant score" and the "phenotype score". The former indicated variant rarity and pathogenicity, and the latter represented the potential clinical relevance of the gene harboring the variant. The latest PhenIX 1.16 was integrated into the Exomiser software suite and shared some functional frameworks with Exomiser^3^. In this study, PhenIX was run within the Exomiser software suite.

AMELIE^4^ used natural language processing (NLP) to construct a homogeneous knowledge base storing parsing results of a huge amount of articles in PubMed. A score was assigned to measure the matching degree between the queried HPO terms and HPO terms extracted from the literature, for each matched literature of a queried gene (variant). Given that the output result of the latest version 3.1.0 (<https://amelie.stanford.edu/>) gives an unsorted result gene list, in this study, the maximum value of the scores of each candidate gene was used as an index for gene ranking, and tied indexes were broken by average value. The general-purpose application program interface (API) of this online prioritizer was utilized to acquire ranking results in batch.

LIRICAL^5^ exploited the likelihood ratio (LR) statistical framework for causal-gene prioritization. It provided an estimated posttest probability of each candidate's diagnosis and allowed clinicians to evaluate the contribution of each phenotypic abnormality to each candidate's diagnosis. LIRICAL used data files from Exomiser for VCF annotation and variant interpretation. The latest stable version 1.3.4 (<https://github.com/TheJacksonLaboratory/LIRICAL/releases>) was adopted in this work.

# Section S2 Characteristics of the DDD and KGD trio cohort

The distributions of HPO and variant amounts of probands of the DDD and KGD trio cohort were shown in **Supplemental Figure 1A** and **2B.** And the sex and age distributions were displayed in **Supplemental Figure 1C** and **2D**. In the DDD trio cohort, the proportions of males and females were close (46.2% vs 53.8%) while in the KGD trio cohort, males were predominant (63.2%). The ages of DDD patients were mainly categorized into the group age 1-7 (48.5%) and 7-18 (50.5%). The age distribution of KGD patients looked like a staircase with the group of ages 0-1 (43.4%) as the top step (**Supplemental Figure 1D**).

The amounts of the unique causal genes of the DDD and KGD cohort were 157 and 110 respectively (**Appendix I**). As with the frequency of causal genes, 25 genes showed recurring appearances with a frequency > 3 each in all 458 patients of the two cohorts (**Supplemental Figure 1E**). Specifically, the high-frequency (> 5) causal genes in DDD cohort included *MED13L* (8), *KCNQ2* (6), *ARID1B* (11), *SCN2A* (6), *SYNGAP1* (7), *SATB2* (6) and *ANKRD11* (7). In the KGD cohort, *SRD5A2* (7) and *KCNQ2* (6) were the prominent genes with the most occurrences, and the latter was the only one standing out in both cohorts. More than half of the patients (161/305) in the DDD cohort were characterized by global developmental delay (HP:0001263) (**Supplemental Figure 1F**). Other common phenotypes (frequency > 30) in this cohort included delayed speech and language development (HP:0000750, 53), microcephaly (HP:0000252, 53), seizure (HP:0001250, 51), and intellectual disability (HP:0001249, 43). In the KGD cohort, seizure (HP:0001250, 35), global developmental delay (HP:0001263, 16), motor delay (HP:0001270, 12), and language development (HP:0000750, 11) were the top four most frequent (>10) disorders (**Supplemental Figure 1F**, **Appendix I**).

If using parent-class HPO terms to characterize patients, 93% of cases of the DDD cohort were shown to carry abnormality of the nervous system (HP:0000707, 284) (**Supplemental Figure 1G**). Besides, 75% and 71% had an abnormality of the musculoskeletal system (HP:0033127, 229) and an abnormality of the head or neck (HP:0000152, 217) respectively. All other congenital abnormalities happened in less than 50% of the cohort. Same to the DDD cohort, abnormality of the nervous system (HP:0000707, 71) and abnormality of the musculoskeletal system (HP:0033127, 39) were the two main features in the KGD cohort (**Supplemental Figure 1G**). Moreover, abnormality of metabolism/homeostasis (HP:0001939, 32) and abnormality of the genitourinary system (HP:0000119, 31) were observed in about one-fifth of the in-house cohort. The definitions of HPO terms in **Supplemental Figure 1F** and **1G** were included in **Supplemental Table S1**. 15 co-occurrence pairs of parent HPO terms were identified in the DDD cohort (**Supplemental Table S2**) and HP:0040064 (Abnormality of limbs) was the most common partner with five pairings. Three co-occurrence pairs and three mutual exclusivity pairs were also discovered in the KGD cohort.

**
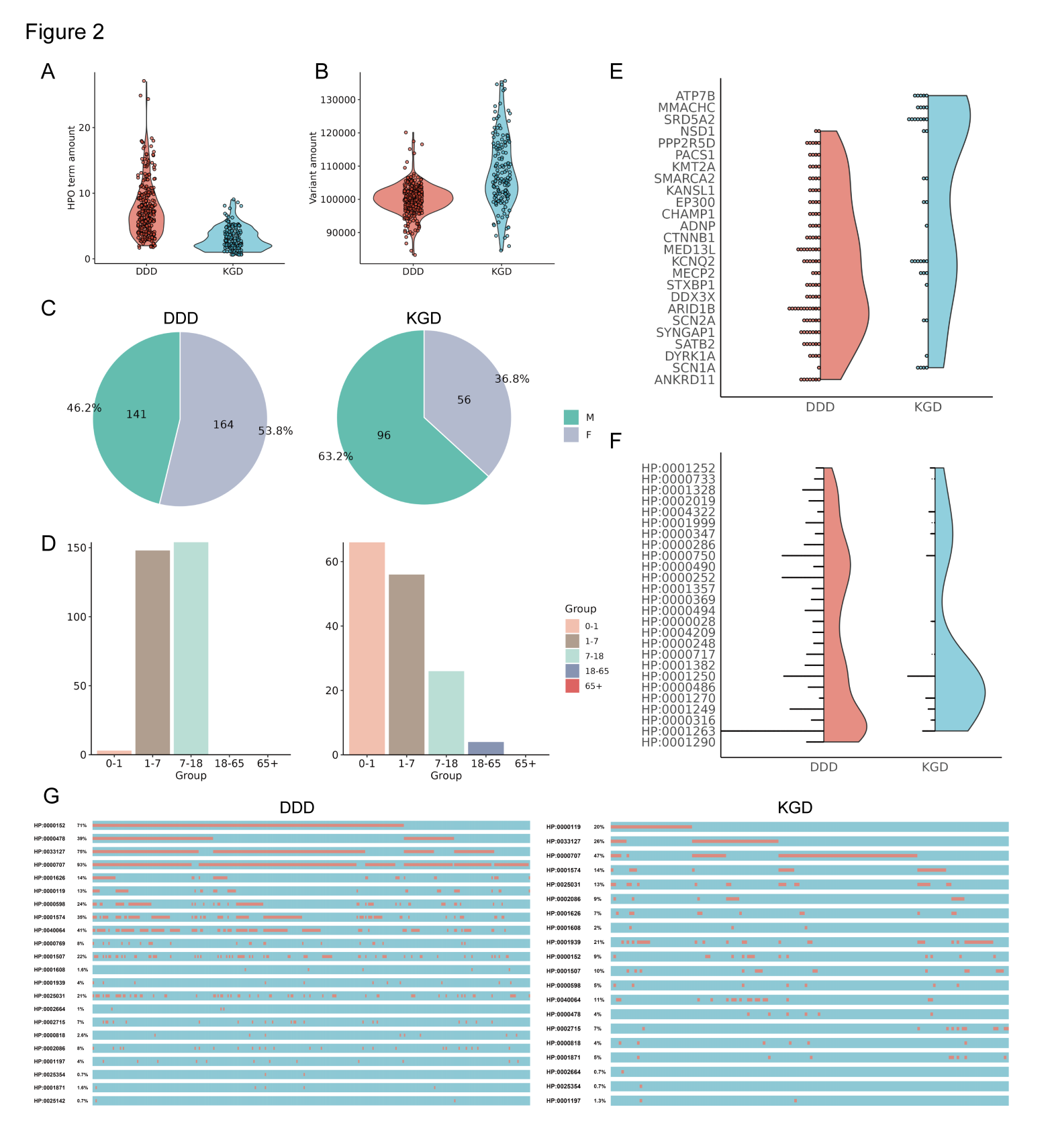
Supplemental Figure 1** **Visualization of characteristics of two trio cohorts used for this assessment.**

**(A)** and **(B)** The distributions of proband HPO and variant amount of DDD **(left)** and KGD **(right)** cohort. **(C)** and **(D)** The proportions of proband sex and age of both cohorts. **(E)** and **(F)** The frequencies of recurring causal genes and HPO terms of both cohorts. **(G)** The distributions of parent-class HPO terms of both cohorts. The definitions of HPO terms in **(F)** and **(G)** were included in **Supplemental Table S1.**

**Supplemental Table S1 The definitions of HPO terms in Supplemental Figure 1F and 1G**

|  | HPO term | Definition |
| --- | --- | --- |
| Supplemental Figure 1F | HP:0001252 | Hypotonia |
|  | HP:0000733 | Abnormal repetitive mannerisms |
|  | HP:0001328 | Specific learning disability |
|  | HP:0002019 | Constipation |
|  | HP:0004322 | Short stature |
|  | HP:0001999 | Abnormal facial shape |
|  | HP:0000347 | Micrognathia |
|  | HP:0000286 | Epicanthus |
|  | HP:0000750 | Delayed speech and language development |
|  | HP:0000490 | Deeply set eye |
|  | HP:0000252 | Microcephaly |
|  | HP:0001357 | Plagiocephaly |
|  | HP:0000369 | Low-set ears |
|  | HP:0000494 | Downslanted palpebral fissures |
|  | HP:0000028 | Cryptorchidism |
|  | HP:0004209 | Clinodactyly of the 5th finger |
|  | HP:0000248 | Brachycephaly |
|  | HP:0000717 | Autism |
|  | HP:0001382 | Joint hypermobility |
|  | HP:0001250 | Seizure |
|  | HP:0000486 | Strabismus |
|  | HP:0001270 | Motor delay |
|  | HP:0001249 | Intellectual disability |
|  | HP:0000316 | Hypertelorism |
|  | HP:0001263 | Global developmental delay |
|  | HP:0001290 | Generalized hypotonia |
| Supplemental Figure 1G left | HP:0000152 | Abnormality of head or neck |
|  | HP:0000478 | Abnormality of the eye |
|  | HP:0033127 | Abnormality of the musculoskeletal system |
|  | HP:0000707 | Abnormality of the nervous system |
|  | HP:0001626 | Abnormality of the cardiovascular system |
|  | HP:0000119 | Abnormality of the genitourinary system |
|  | HP:0000598 | Abnormality of the ear |
|  | HP:0001574 | Abnormality of the integument |
|  | HP:0040064 | Abnormality of limbs |
|  | HP:0000769 | Abnormality of the breast |
|  | HP:0001507 | Growth abnormality |
|  | HP:0001608 | Abnormality of the voice |
|  | HP:0001939 | Abnormality of metabolism/homeostasis |
|  | HP:0025031 | Abnormality of the digestive system |
|  | HP:0002664 | Neoplasm |
|  | HP:0002715 | Abnormality of the immune system |
|  | HP:0000818 | Abnormality of the endocrine system |
|  | HP:0002086 | Abnormality of the respiratory system |
|  | HP:0001197 | Abnormality of prenatal development or birth |
|  | HP:0025354 | Abnormal cellular phenotype |
|  | HP:0001871 | Abnormality of blood and blood-forming tissues |
|  | HP:0025142 | Constitutional symptom |
| Supplemental Figure 1G right | HP:0000119 | Abnormality of the genitourinary system |
|  | HP:0033127 | Abnormality of the musculoskeletal system |
|  | HP:0000707 | Abnormality of the nervous system |
|  | HP:0001574 | Abnormality of the integument |
|  | HP:0025031 | Abnormality of the digestive system |
|  | HP:0002086 | Abnormality of the respiratory system |
|  | HP:0001626 | Abnormality of the cardiovascular system |
|  | HP:0001608 | Abnormality of the voice |
|  | HP:0001939 | Abnormality of metabolism/homeostasis |
|  | HP:0000152 | Abnormality of head or neck |
|  | HP:0001507 | Growth abnormality |
|  | HP:0000598 | Abnormality of the ear |
|  | HP:0040064 | Abnormality of limbs |
|  | HP:0000478 | Abnormality of the eye |
|  | HP:0002715 | Abnormality of the immune system |
|  | HP:0000818 | Abnormality of the endocrine system |
|  | HP:0001871 | Abnormality of blood and blood-forming tissues |
|  | HP:0002664 | Neoplasm |
|  | HP:0025354 | Abnormal cellular phenotype |
|  | HP:0001197 | Abnormality of prenatal development or birth |

**Supplemental Table S2 Results of co-occurrence and mutual exclusivity analysis based on parent-class HPO terms of DDD and KGD cases**

|  | A | B | Neither | A Not B | B Not A | Both | p-Value | q-Value | Tendency |
| --- | --- | --- | --- | --- | --- | --- | --- | --- | --- |
| DDD | HP:0033127 | HP:0040064 | 70 | 110 | 6 | 119 | <0.001 | <0.001 | Co-occurrence |
|  | HP:0000152 | HP:0001574 | 78 | 119 | 10 | 98 | <0.001 | <0.001 | Co-occurrence |
|  | HP:0000152 | HP:0033127 | 39 | 37 | 49 | 180 | <0.001 | <0.001 | Co-occurrence |
|  | HP:0002715 | HP:0002086 | 269 | 12 | 15 | 9 | <0.001 | <0.001 | Co-occurrence |
|  | HP:0000598 | HP:0001574 | 166 | 31 | 67 | 41 | <0.001 | <0.001 | Co-occurrence |
|  | HP:0000152 | HP:0040064 | 68 | 112 | 20 | 105 | <0.001 | <0.001 | Co-occurrence |
|  | HP:0001574 | HP:0040064 | 132 | 48 | 65 | 60 | <0.001 | 0.004 | Co-occurrence |
|  | HP:0000119 | HP:0025031 | 219 | 22 | 46 | 18 | <0.001 | 0.005 | Co-occurrence |
|  | HP:0000598 | HP:0040064 | 151 | 29 | 82 | 43 | <0.001 | 0.005 | Co-occurrence |
|  | HP:0025354 | HP:0001871 | 300 | 0 | 3 | 2 | <0.001 | 0.005 | Co-occurrence |
|  | HP:0000152 | HP:0000598 | 78 | 155 | 10 | 62 | <0.001 | 0.015 | Co-occurrence |
|  | HP:0040064 | HP:0000769 | 174 | 108 | 6 | 17 | <0.001 | 0.018 | Co-occurrence |
|  | HP:0001939 | HP:0025354 | 294 | 9 | 0 | 2 | 0.001 | 0.021 | Co-occurrence |
|  | HP:0001574 | HP:0025031 | 166 | 75 | 31 | 33 | 0.002 | 0.036 | Co-occurrence |
|  | HP:0001626 | HP:0002664 | 263 | 39 | 0 | 3 | 0.002 | 0.038 | Co-occurrence |
| KGD | HP:0033127 | HP:0040064 | 110 | 25 | 3 | 14 | <0.001 | <0.001 | Co-occurrence |
|  | HP:0001574 | HP:0025031 | 120 | 12 | 10 | 10 | <0.001 | 0.004 | Co-occurrence |
|  | HP:0000119 | HP:0000707 | 55 | 26 | 66 | 5 | <0.001 | 0.006 | Mutual exclusivity |
|  | HP:0000707 | HP:0001939 | 55 | 65 | 26 | 6 | <0.001 | 0.013 | Mutual exclusivity |
|  | HP:0000707 | HP:0002715 | 70 | 71 | 11 | 0 | <0.001 | 0.027 | Mutual exclusivity |
|  | HP:0002715 | HP:0001871 | 137 | 7 | 4 | 4 | <0.001 | 0.029 | Co-occurrence |

# Section S3 Details of the misjudged cases

KGD Case NP22F4236 was a five-month-old seizure patient who carried a single-base duplication in gene *PRRT2*. This frameshift mutation c.641dupC (p.Arg217Profs*8) introduced a premature translation termination. *PRRT2*-associated neurological disorders shared autosomal dominant inheritance but exhibited incomplete penetrance^6-14^. This explained that the baby patient’s disease-causing variant derived from his mother while his mother was healthy. Exomiser in singleton mode ranked *PRRT2* in the 3rd and 1st place respectively under Protocols A and B. In trio mode (Protocol C and D), *PRRT2* was thoroughly ruled out according to the laws of inheritance.

KGD Case NP22FW1164 carried a paternal nonsense variant in gene *DEPDC5*. The pathogenic variant c.1663C>T (p.Arg555Ter) could result in protein truncation or nonsense mediated decay. Same as the situation of case NP22F4236, Exomiser in trio mode misjudged this case due to disease incomplete penetrance^15-22^: the father carrier was free of febrile seizure.

The causal variant of DDD case DDDP110879 was located in gene *GJB2*, an essential gene for genetic deafness or hearing impairment. The ClinGen Hearing Loss Variant Curation Expert Panel held that the pathogenic evidence of this variant outweighed its high AF. Based on the Updated Recommendation for the Benign Stand Alone ACMG/AMP Criterion^23^, this variant could be exempted from the benign rule BA1 in pathogenicity classification.

As with KGD case NP23FW3882 affected by mutated *PRRT2*, the max AF of the causal variant in gnomAD was 0.96%, which resulted in the failed diagnosis by AMELIE in default state.

KGD Case NP24FW2307 suffered from jaundice and talipes equinovarus, and the former was the most typical clinical manifestation of glucose-6-phosphate dehydrogenase (G6PD) deficiency. This deficiency was an X-linked recessive disease, and a common genetic disorder in China with an overall prevalence of 2.1%^24^. The two-year-old male patient carried a c.1388G>A (p.Arg463His) variant, a hotspot for G6PD deficiency in the Chinese population^24-29^. However, the variant AF was a little high in the "healthy" East Asian population. As described in OMIM, most G6PD-deficient patients were asymptomatic throughout their life.

# Section S4 Performance alteration measurement for Exomiser along with minor factor adjustment

The Exomiser parameter option named Failedvariantfilter (FVF) helped to remove low-quality variants which were not flagged as PASS or "." in the FILTER column of a VCF file. This filtering function was highly recommended by the authors of Exomiser and was switched on by default in version 13.1.0 used in this study. Nevertheless, this option was not activated in advance in the old version 12.1.0 evaluated in our prior study^30^. In order to establish a connection with our early work to systematically weigh the effect of certain parameters or factors, the old Exomiser 12.1.0 (database version: 2102) was reused herein. Exomiser 12.1.0 with FVF activated ranked the true gene in top-1, and within top-5, -10, -20, -30, -40, and -50 for about 26.6, 60.7, 75.7, 87.2, 91.8, 94.4 and 95.1% of the total cases (**Supplemental Table S3** Row 3), increasing by 11.5, 22.3, 22.6, 16.7, 12.8, 10.5 and 8.5%, respectively in contrast with Exomiser 12.1.0 in the default state (**Supplemental Table S3** Row 2) which was benchmarked in our previous research^30^. It should be pointed out that the FVF option was one of the primary reasons leading to the result discrepancy between our prior work and another report^31,32^.

The "frequencySources" parameter was also adjusted to investigate its effect on Exomiser. The UK10K and the non-Finnish European population of gnomAD (GNOMAD_E_NFE and GNOMAD_G_NFE) were selected as the population AF references for DDD cases which all came from the United Kingdom. Instead of the default frequency sources involving dozens of items, the subtraction in the AF reference completely undermined the power of Exomiser (**Supplemental Table S3** Row 1). One of the reasons might be the "impurity" of the UK10K and the non-Finnish European population. Moreover, the phenomenon raises the possibility that the performance could be enhanced if conversely, a user adds other population AF sources, especially those derived from the local population to the default frequency sources.

The performance alteration caused by the updates of software and its attached database was disclosed as well: the sensitivity was elevated across all top-level experiments in Exomiser 13.1.0 in the default state (**Supplemental Table S3** Row 4, database version: 2209) in contrast with Exomiser 12.1.0 with FVF activated (**Supplemental Table S3** Row 3, database version: 2102).

Taking the statistics of Exomiser 13.1.0 under protocol D (**Supplemental Table S3** Row 5) as the peak value, the overall sensitivity of Exomiser increased incrementally along with parameter optimizations or updates of software and its attached database. The causal-gene ranking trends for Exomiser along with minor factor adjustment were recorded in **Appendix II**.

**Supplemental Table S3 Sensitivity of Exomiser under different versions and/or parameter settings in each top-level experiment assessed using the DDD dataset.**

|  | top1(%) | top5(%) | top10(%) | top20(%) | top30(%) | top40(%) | top50(%) |
| --- | --- | --- | --- | --- | --- | --- | --- |
| V12.1 SUB.AF | 13.8 | 34.8 | 48.9 | 68.2 | 75.1 | 82 | 84.9 |
| V12.1 default | 15.1 | 38.4 | 53.1 | 70.5 | 79 | 83.9 | 86.6 |
| V12.1 FVF On | 26.6 | 60.7 | 75.7 | 87.2 | 91.8 | 94.4 | 95.1 |
| V13.1 PROT.A | 31.8 | 73.1 | 85.2 | 92.8 | 94.4 | 95.4 | 96.1 |
| V13.1 PROT.D | 61.6 | 86.6 | 91.1 | 95.1 | 96.7 | 97.4 | 97.4 |

V12.1 SUB.AF represents Exomiser 12.1.0 with subtracted population AF sources.

V12.1 default represents Exomiser 12.1.0 in default settings.

V12.1 FVF On represents Exomiser 12.1.0 with Failedvariantfilter option enabled.

V13.1 PROT.A and V13.1 PROT.D represents Exomiser 13.1.0 under Protocol A and D respectively.

# Section S5 Comparison of prioritizer performance assessment results from different studies

Tosco-Herrera et al. benchmarked causal-gene prioritization tools with WES data of 61 unrelated singleton cases^33^. To make a comparison with their study, the average value of the sensitivity of the DDD and KGD cohort (**Main Text** **Table 1**) was calculated for each of the four software under optimum conditions. In the top-1 experiment setting, the performance between our study and Tosco-Herrera's is close for Exomiser and AMELIE (**Supplemental Table S4**). In the top-10 setting, all four software have better achievements in our study than that reported by Tosco-Herrera et al. The utilization of trio mode and software version differences might account for most of the discrepancies. It should be noted that in Tosco-Herrera's study, parameter configurations of the prioritizers were not described.

**Supplemental Table S4 Comparison of prioritizer performance assessment results from different studies**

|  | Yuan et al. | Tosco-H et al. | Yuan et al. | Tosco-H et al. | Yuan et al. | Tosco-H et al. |
| --- | --- | --- | --- | --- | --- | --- |
|  | top1(%) | | top5(%) | | top10(%) | |
| Exomiser | 65 | 68.8 | 86.1 | 75.4 | 90.3 | 78.7 |
| PhenIX | 59.3 | 67.2 | 83.1 | 72.1 | 91 | 78.7 |
| AMELIE | 49.3 | 47.5 | 80.8 | 70.5 | 86.7 | 73.8 |
| LIRICAL | 52.6 | 65.6 | 75.6 | 77 | 84.4 | 78.7 |

# Reference

1 Smedley, D. *et al.* Next-generation diagnostics and disease-gene discovery with the Exomiser. *Nature protocols* **10**, 2004-2015 (2015).

2 Zemojtel, T. *et al.* Effective diagnosis of genetic disease by computational phenotype analysis of the disease-associated genome. *Sci Transl Med* **6**, 252ra123, doi:10.1126/scitranslmed.3009262 (2014).

3 Kelly, C. *et al.* Phenotype-aware prioritisation of rare Mendelian disease variants. *Trends Genet* **38**, 1271-1283, doi:10.1016/j.tig.2022.07.002 (2022).

4 Birgmeier, J. *et al.* AMELIE speeds Mendelian diagnosis by matching patient phenotype and genotype to primary literature. *Science translational medicine* **12** (2020).

5 Robinson, P. N. *et al.* Interpretable clinical genomics with a likelihood ratio paradigm. *The American Journal of Human Genetics* **107**, 403-417 (2020).

6 Schubert, J. *et al.* PRRT2 mutations are the major cause of benign familial infantile seizures. *Hum Mutat* **33**, 1439-1443, doi:10.1002/humu.22126 (2012).

7 Li, J. *et al.* Targeted genomic sequencing identifies PRRT2 mutations as a cause of paroxysmal kinesigenic choreoathetosis. *J Med Genet* **49**, 76-78, doi:10.1136/jmedgenet-2011-100635 (2012).

8 Friedman, J., Olvera, J., Silhavy, J. L., Gabriel, S. B. & Gleeson, J. G. Mild paroxysmal kinesigenic dyskinesia caused by PRRT2 missense mutation with reduced penetrance. *Neurology* **79**, 946-948, doi:10.1212/WNL.0b013e318266fabf (2012).

9 Liu, X. R. *et al.* Novel PRRT2 mutations in paroxysmal dyskinesia patients with variant inheritance and phenotypes. *Genes Brain Behav* **12**, 234-240, doi:10.1111/gbb.12008 (2013).

10 Zhang, L. M. *et al.* Reduced Penetrance of PRRT2 Mutation in a Chinese Family With Infantile Convulsion and Choreoathetosis Syndrome. *J Child Neurol* **30**, 1263-1269, doi:10.1177/0883073814556887 (2015).

11 Chen, G. H. Five cases of paroxysmal kinesigenic dyskinesia by genetic diagnosis. *Exp Ther Med* **9**, 909-912, doi:10.3892/etm.2014.2155 (2015).

12 Vlaskamp, D. R. M. *et al.* PRRT2-related phenotypes in patients with a 16p11.2 deletion. *Eur J Med Genet* **62**, 265-269, doi:10.1016/j.ejmg.2018.08.002 (2019).

13 Balagura, G. *et al.* Clinical spectrum and genotype-phenotype correlations in PRRT2 Italian patients. *Eur J Paediatr Neurol* **28**, 193-197, doi:10.1016/j.ejpn.2020.06.005 (2020).

14 Yang, L. *et al.* Novel and de novo point and large microdeletion mutation in PRRT2-related epilepsy. *Brain Behav* **10**, e01597, doi:10.1002/brb3.1597 (2020).

15 Lal, D. *et al.* DEPDC5 mutations in genetic focal epilepsies of childhood. *Ann Neurol* **75**, 788-792, doi:10.1002/ana.24127 (2014).

16 Zhang, X. *et al.* Phenotypic and Genotypic Characterization of DEPDC5-Related Familial Focal Epilepsy: Case Series and Literature Review. *Front Neurol* **12**, 641019, doi:10.3389/fneur.2021.641019 (2021).

17 Tsai, M. H. *et al.* DEPDC5 mutations in familial and sporadic focal epilepsy. *Clin Genet* **92**, 397-404, doi:10.1111/cge.12992 (2017).

18 Picard, F. *et al.* DEPDC5 mutations in families presenting as autosomal dominant nocturnal frontal lobe epilepsy. *Neurology* **82**, 2101-2106, doi:10.1212/WNL.0000000000000488 (2014).

19 Ishida, S. *et al.* Mutations of DEPDC5 cause autosomal dominant focal epilepsies. *Nat Genet* **45**, 552-555, doi:10.1038/ng.2601 (2013).

20 Dibbens, L. M. *et al.* Mutations in DEPDC5 cause familial focal epilepsy with variable foci. *Nat Genet* **45**, 546-551, doi:10.1038/ng.2599 (2013).

21 Martin, C. *et al.* A recurrent mutation in DEPDC5 predisposes to focal epilepsies in the French-Canadian population. *Clin Genet* **86**, 570-574, doi:10.1111/cge.12311 (2014).

22 Scheffer, I. E. *et al.* Mutations in mammalian target of rapamycin regulator DEPDC5 cause focal epilepsy with brain malformations. *Ann Neurol* **75**, 782-787, doi:10.1002/ana.24126 (2014).

23 Ghosh, R. *et al.* Updated recommendation for the benign stand-alone ACMG/AMP criterion. *Hum Mutat* **39**, 1525-1530, doi:10.1002/humu.23642 (2018).

24 He, Y. *et al.* Glucose-6-phosphate dehydrogenase deficiency in the Han Chinese population: molecular characterization and genotype-phenotype association throughout an activity distribution. *Sci Rep* **10**, 17106, doi:10.1038/s41598-020-74200-y (2020).

25 Peng, Q. *et al.* Large cohort screening of G6PD deficiency and the mutational spectrum in the Dongguan District in Southern China. *PLoS One* **10**, e0120683, doi:10.1371/journal.pone.0120683 (2015).

26 Lin, F., Lou, Z. Y., Xing, S. Y., Zhang, L. & Yang, L. Y. The gene spectrum of glucose-6-phosphate dehydrogenase (G6PD) deficiency in Guangdong province, China. *Gene* **678**, 312-317, doi:10.1016/j.gene.2018.07.068 (2018).

27 Chang, J. G. *et al.* Molecular characterization of glucose-6-phosphate dehydrogenase (G6PD) deficiency by natural and amplification created restriction sites: five mutations account for most G6PD deficiency cases in Taiwan. *Blood* **80**, 1079-1082 (1992).

28 Ainoon, O. *et al.* Glucose-6-phosphate dehydrogenase (G6PD) variants in Malaysian Chinese. *Hum Mutat* **14**, 352, doi:10.1002/(SICI)1098-1004(199910)14:4<352::AID-HUMU14>3.0.CO;2-G (1999).

29 Saha, S. *et al.* Molecular characterisation of red cell glucose-6-phosphate dehydrogenase deficiency in Singapore Chinese. *Am J Hematol* **47**, 273-277, doi:10.1002/ajh.2830470405 (1994).

30 Yuan, X. *et al.* Evaluation of phenotype-driven gene prioritization methods for Mendelian diseases. *Brief Bioinform* **23**, doi:10.1093/bib/bbac019 (2022).

31 Jacobsen, J. O. B., Kelly, C., Cipriani, V., Robinson, P. N. & Smedley, D. Evaluation of phenotype-driven gene prioritization methods for Mendelian diseases. *Brief Bioinform* **23**, doi:10.1093/bib/bbac188 (2022).

32 Yuan, X. & Zhang, P. Revisiting benchmark study for response to methodological critiques of 'Evaluation of phenotype-driven gene prioritization methods for Mendelian diseases'. *Brief Bioinform* **23**, doi:10.1093/bib/bbac181 (2022).

33 Tosco-Herrera, E. *et al.* Evaluation of a whole-exome sequencing pipeline and benchmarking of causal germline variant prioritizers. *Hum Mutat* **43**, 2010-2020, doi:10.1002/humu.24459 (2022).

# Appendix I Information of HPO term, causal gene, age, sex, and parental disease status of each case in the KGD cohort

| KGD ID | HPO term | Gene | Age | Sex | Dad affected? | Mum affected? |
| --- | --- | --- | --- | --- | --- | --- |
| NP21F0972 | HP:0000054 | SRD5A2 | 11Y | M | no | no |
| NP21F2197 | HP:0001281 | KCNQ2 | 36D | F | no | no |
| NP21F4007 | HP:0000750 HP:0000726 | SHANK3 | 3Y8M | F | no | no |
| NP21F4924 | HP:0001250 HP:0001270 | KCNB1 | 10M | M | no | no |
| NP21F4975 | HP:0000953 HP:0001249 HP:0001263 HP:0001251 | XPA | 7Y | M | no | no |
| NP21F5381 | HP:0008935 HP:0008752 HP:0002033 | RYR1 | 30D | M | no | no |
| NP21F5728 | HP:0003248 | SRD5A2 | 7M | M | no | no |
| NP21F6023 | HP:0000952 HP:0001643 HP:0000126 HP:0001612 | SETBP1 | 6D | F | no | no |
| NP21F6192 | HP:0030203 | BCHE | 1Y | M | no | no |
| NP21F6186 | HP:0001943 HP:0001262 HP:0001281 HP:0002904 | MMACHC | 39D | F | no | no |
| NP21F6193 | HP:0001250 | SCN2A | 10D | M | no | no |
| NP21F6382 | HP:0001263 HP:0000347 HP:0000581 HP:0003808 | SMARCA2 | 9M | M | no | no |
| NP21F7203 | HP:0001250 | KCNQ2 | 12D | M | no | no |
| NP21F7355 | HP:0002780 HP:0001631 HP:0001643 | CHD7 | 2M | F | no | no |
| NP22F1260 | HP:0001250 | DNM1L | 7Y | M | no | no |
| NP22F2042 | HP:0001250 | SCN8A | 7M | F | no | no |
| NP22F2828 | HP:0002748 HP:0004322 HP:0000924 | COL2A1 | 30Y | M | no | no |
| NP22F2862 | HP:0001305 HP:0002094 HP:0002032 HP:0002575 HP:0008572 HP:0000028 | CHD7 | 8D | M | no | no |
| NP22F2948 | HP:0002912 | MMACHC | 51D | M | no | no |
| NP22F3020 | HP:0000316 HP:0000422 HP:0008050 HP:0000954 HP:0005989 | SOS1 | 3D | F | no | no |
| NP22F3138 | HP:0004323 HP:0000953 HP:0000811 | CYP21A2 | 20D | F | no | no |
| NP22F3895 | HP:0033148 HP:0001939 | IVD | 10D | M | no | no |
| NP22F3888 | HP:0001250 HP:0001317 HP:0002015 HP:0002033 HP:0002020 | SUOX | 1D | M | no | no |
| NP22F4156 | HP:0006644 HP:0000765 HP:0002094 | DYNC2H1 | 14D | F | no | no |
| NP22F4236 | HP:0001250 | PRRT2 | 5M | M | no | no |
| NP22F4233 | HP:0002094 HP:0000347 HP:0001238 HP:0005060 HP:0001612 | CRLF1 | 5D | M | no | no |
| NP22F4768 | HP:0000952 HP:0001250 | OTC | 7D | M | no | no |
| NP22F4952 | HP:0002721 HP:0000155 | DCLRE1C | 33D | M | no | no |
| NP22F5410 | HP:0001263 HP:0001646 | ZEB2 | 5Y | M | no | no |
| NP22F6401 | HP:0002333 HP:0000750 | TH | 8M | M | no | no |
| NP22F6580 | HP:0010837 | ATP7B | 13Y | M | no | no |
| NP22F6941 | HP:0000787 | CLDN16 | 5Y | M | yes | no |
| NP22F7266 | HP:0000826 HP:0001161 | GLI3 | 11M | M | no | no |
| NP22F7394 | HP:0001263 | ADNP | 9Y | M | no | no |
| NP22F7986 | HP:0002900 | SLC12A3 | 1Y5M | M | no | no |
| NP22F8675 | HP:0007210 | DYSF | 27Y | F | no | no |
| NP22F8941 | HP:0001263 HP:0002373 | MEF2C | 1Y | F | no | no |
| NP22FW0080 | HP:0002955 | CYBB | 7M | M | no | no |
| NP22FW0166 | HP:0001250 | SLC13A5 | 29D | F | no | no |
| NP22FW0161 | HP:0001319 | EXOSC3 | 1D | F | no | no |
| NP22FW0217 | HP:0002910 HP:0010837 | ATP7B | 15Y | M | no | no |
| NP22FW0245 | HP:0000787 HP:0010474 | SLC3A1 | 10Y | M | no | no |
| NP22FW0379 | HP:0002900 | BSND | 82D | M | no | no |
| NP22FW0478 | HP:0000572 HP:0001288 HP:0002415 | ABCD1 | 6Y | M | no | no |
| NP22FW0437 | HP:0000051 | SRD5A2 | 2Y | M | no | no |
| NP22FW0520 | HP:0001250 HP:0000750 HP:0001270 | SMARCA2 | 1Y | M | no | no |
| NP22FW0580 | HP:0000924 HP:0001627 HP:0001238 | FBN1 | 1D | F | no | no |
| NP22FW0567 | HP:0002910 | ATP7B | 3Y | F | no | no |
| NP22FW0606 | HP:0000787 | GRHPR | 1Y | M | no | no |
| NP22F8064 | HP:0002315 HP:0001250 | ATP7B | 13Y | M | no | no |
| NP22FW0040 | HP:0001249 HP:0002378 | DUOX2 | 10Y | F | no | yes |
| NP22FW0774 | HP:0001399 HP:0006562 | ATP7B | 4Y | M | no | no |
| NP22FW0844 | HP:0003265 HP:0001392 HP:0000952 HP:0001433 | MMUT | 3D | F | no | no |
| NP22FW0893 | HP:0000047 HP:0000054 | SRD5A2 | 4Y | M | no | no |
| NP22FW0975 | HP:0001396 HP:0001943 HP:0001905 HP:0000821 HP:0000085 HP:0003270 HP:0001541 HP:0006476 HP:0004360 | BCS1L | 26D | M | no | no |
| NP22FW1018 | HP:0003131 HP:0003159 | SLC3A1 | 4Y | M | yes | no |
| NP22FW1153 | HP:0001875 HP:0002090 HP:0003265 HP:0001655 | ELANE | 16D | M | yes | no |
| NP22FW1164 | HP:0001250 HP:0002373 | DEPDC5 | 7Y | F | no | no |
| NP23FW2438 | HP:0003236 HP:0032232 HP:0002910 | DMD | 5D | M | no | no |
| NP23FW2594 | HP:0000047 HP:0000119 HP:0000795 HP:0000032 | AR | 1Y | M | no | no |
| NP23FW2755 | HP:0000787 HP:0008672 HP:0010474 | HOGA1 | 7M | M | no | no |
| NP23FW2748 | HP:0001263 HP:0001272 HP:0001321 HP:0001317 HP:0001270 HP:0006895 HP:0000750 HP:0001249 | KIF1A | 2Y | F | no | no |
| NP23FW3016 | HP:0000952 HP:0003265 HP:0006579 HP:0011985 | DGUOK | 16D | M | no | no |
| NP23FW3511 | HP:0002373 HP:0001281 HP:0003739 HP:0001250 HP:0011097 HP:0001263 HP:0001270 | COL4A1 | 4Y | F | no | no |
| NP23FW3619 | HP:0001531 HP:0001510 HP:0001507 HP:0002151 HP:0002902 HP:0002900 HP:0200114 HP:0003113 | CFTR | 6M | F | no | no |
| NP23FW4138 | HP:0000047 HP:0008743 HP:0000054 HP:0008736 HP:0030260 HP:0100587 | SRD5A2 | 13Y | M | no | no |
| NP23FW4197 | HP:0004357 HP:0010911 HP:0010910 HP:0010914 | BCKDHB | 19D | M | no | no |
| NP23FW4254 | HP:0000961 HP:0002094 HP:0004886 HP:0006511 HP:0011675 HP:0001692 HP:0010967 HP:0000364 HP:0000036 | HRAS | 15D | M | no | no |
| NP23FW4527 | HP:0000032 HP:0000033 HP:0000037 HP:0000050 HP:0012861 | AR | 8M | M | no | no |
| NP23FW4693 | HP:0000035 HP:0008715 HP:0008733 HP:0000028 HP:0008689 | ANOS1 | 12Y | M | no | no |
| NP23FW4819 | HP:0000790 HP:0000787 HP:0030038 HP:0000126 HP:0008672 | AGXT | 15Y | M | no | no |
| NP24FW0344 | HP:0000062 HP:0000032 HP:0000050 HP:0000051 HP:0000033 | SRD5A2 | 2D | M | no | no |
| NP23FW0026 | HP:0001987 HP:0008315 HP:0045045 HP:0001622 HP:0001943 HP:0001511 HP:0000054 | SLC25A20 | 7D | M | no | no |
| NP23FW0093 | HP:0003623 HP:0000952 HP:0002045 HP:0002153 | MMACHC | 18D | M | no | no |
| NP23FW0138 | HP:0001270 HP:0000998 HP:0000953 HP:0001410 HP:0045056 HP:0008606 | TFAP2A | 4M | M | no | no |
| NP23FW0230 | HP:0000316 HP:0000357 HP:0001612 HP:0003401 | EBF3 | 3D | M | no | no |
| NP23FW0238 | HP:0000033 HP:0000795 | AR | 11Y | M | no | no |
| NP23FW0412 | HP:0001250 HP:0002353 | PCDH19 | 1Y | F | no | no |
| NP23FW0509 | HP:0001762 HP:0001220 HP:0012768 | PIEZO2 | 5D | M | no | no |
| NP23FW0520 | HP:0000347 HP:0001998 HP:0001511 HP:0000364 | SF3B4 | 4D | F | no | no |
| NP23FW0524 | HP:0000790 HP:0000123 | COL4A4 | 15Y | M | no | yes |
| NP23FW0574 | HP:0001263 HP:0001250 HP:0001249 | KCNQ2 | 7Y | F | no | no |
| NP23FW0746 | HP:0001891 HP:0008153 HP:0100512 | ACADSB | 26Y | F | no | no |
| NP23FW0769 | HP:0002643 HP:0002090 HP:0002779 HP:0000364 HP:0002153 HP:0001903 | COL2A1 | 7D | F | no | no |
| NP23FW1068 | HP:0000074 HP:0000126 HP:0000107 HP:0000126 HP:0002832 | PKD1 | 7Y | M | no | no |
| NP23FW1081 | HP:0000054 | SRD5A2 | 1Y | M | no | no |
| NP23FW1139 | HP:0001263 HP:0000316 HP:0000581 HP:0010490 HP:0000377 | NIPBL | 3Y | F | no | no |
| NP23FW1210 | HP:0000280 HP:0001167 | IDS | 3Y | M | no | no |
| NP23FW1211 | HP:0001022 | TYR | 1D | F | no | no |
| NP23FW1402 | HP:0032660 HP:0001250 | PRRT2 | 1Y | M | yes | no |
| NP23FW1445 | HP:0001250 | SCN1A | 10Y | F | no | no |
| NP23FW1625 | HP:0001270 HP:0000750 HP:0001250 HP:0001276 | PPP3CA | 1Y | M | no | no |
| NP23FW1751 | HP:0001263 HP:0001249 | EP300 | 5Y | M | no | no |
| NP23FW1885 | HP:0001263 HP:0000750 HP:0001270 | APTX | 5Y | M | no | no |
| NP23FW2404 | HP:0008850 HP:0004322 | PCNT | 2Y | F | no | no |
| NP23FW2348 | HP:0002376 HP:0000733 HP:0002353 HP:0002474 | MECP2 | 2Y | F | no | no |
| NP23FW2472 | HP:0001250 HP:0001510 | HNRNPU | 1Y | F | no | no |
| NP23FW2582 | HP:0001510 HP:0000790 HP:0011968 HP:0002118 | HRAS | 3M | F | no | no |
| NP23FW3203 | HP:0000315 HP:0002643 | FGFR2 | 1D | M | no | no |
| NP23FW3242 | HP:0001998 HP:0001281 HP:0003623 | SUOX | 2Y | F | no | no |
| NP23FW3305 | HP:0001249 HP:0003739 HP:0001250 HP:0000750 HP:0000729 | STXBP1 | 3Y | M | no | no |
| NP23FW3627 | HP:0001510 HP:0001270 HP:0004322 HP:0001249 | PTPN11 | 1Y | M | no | no |
| NP23FW3882 | HP:0001250 HP:0032660 | PRRT2 | 7M | M | no | yes |
| NP23FW4118 | HP:0000952 HP:0006956 | NFIA | 21D | F | no | no |
| NP23FW4233 | HP:0001220 HP:0001601 HP:0001631 HP:0011995 HP:0001263 | ZC4H2 | 1Y | F | no | no |
| NP23FW4379 | HP:0025215 HP:0010532 HP:0001069 | CACNA1A | 6Y | M | no | no |
| NP23FW4695 | HP:0002376 HP:0001249 HP:0001250 | MECP2 | 7Y | F | no | no |
| NP23FW4766 | HP:0007475 HP:0007479 | KRT5 | 1D | F | no | no |
| NP23FW4951 | HP:0001250 | SCN1A | 4Y | F | no | no |
| NP23FW5247 | HP:0000717 HP:0007010 HP:0002474 | NLGN3 | 5Y | M | no | no |
| NP23FW5740 | HP:0002715 HP:0002721 | BTK | 6Y | M | no | no |
| NP23FW6272 | HP:0002384 HP:0001281 HP:0001250 | KCNA1 | 20Y | F | no | no |
| NP23FW6289 | HP:0002023 HP:0005218 HP:0011804 HP:0001250 HP:0001252 HP:0001802 HP:0000028 HP:0000036 | PIGN | 10D | M | no | no |
| NP23FW6353 | HP:0001281 HP:0003739 HP:0001336 HP:0011804 HP:0001250 | KCNQ2 | 18D | M | no | no |
| NP23FW6585 | HP:0002902 HP:0001762 HP:0100759 HP:0002094 HP:0011968 | SCN2A | 26D | F | no | no |
| NP24FW0265 | HP:0001270 HP:0000750 | NSD1 | 3Y | F | no | no |
| NP24FW0329 | HP:0000787 HP:0008672 HP:0010474 | AGXT | 5Y | F | no | no |
| NP24FW0491 | HP:0000316 HP:0000324 HP:0000347 HP:0000175 HP:0001762 | SOX9 | 4D | F | no | no |
| NP24FW0616 | HP:0001263 HP:0000750 | DYRK1A | 3Y | F | no | no |
| NP24FW0617 | HP:0001250 HP:0002373 | SCN1A | 2Y | M | no | no |
| NP24FW0693 | HP:0001159 HP:0040326 HP:0001273 HP:0000044 | FGFR1 | 16Y | F | no | no |
| NP24FW0826 | HP:0000953 HP:0000973 HP:0000958 | FERMT1 | 4Y | M | no | no |
| NP24FW0974 | HP:0002719 HP:0031245 HP:0012262 HP:0032975 HP:0002206 HP:0002721 | DIAPH1 | 9Y | M | no | no |
| NP24FW1189 | HP:0000819 HP:0025388 HP:0001873 HP:0001973 HP:0004322 | LRBA | 11Y | F | no | no |
| NP24FW1376 | HP:0000126 HP:0000787 HP:0001945 | HOGA1 | 1Y | F | no | no |
| NP24FW1604 | HP:0002013 HP:0001945 HP:0001252 HP:0001270 HP:0001263 HP:0001254 | GCDH | 11M | F | no | no |
| NP24FW1753 | HP:0001915 HP:0002014 | RPL5 | 1Y | F | no | no |
| NP24FW1871 | HP:0001305 HP:0000750 HP:0001263 HP:0001270 HP:0002079 | DDHD2 | 1Y | M | no | no |
| NP24FW2307 | HP:0000952 HP:0001762 | G6PD | 2Y | M | no | no |
| NP24FW2366 | HP:0001631 HP:0001238 HP:0000028 HP:0001252 HP:0001298 | NSD1 | 16D | M | no | no |
| NP24FW2409 | HP:0001250 HP:0001276 HP:0002353 HP:0001626 | KCNQ2 | 17D | M | no | no |
| NP24FW2443 | HP:0005599 | TYR | 44D | F | no | no |
| NP24FW2729 | HP:0009381 HP:0000768 HP:0002650 HP:0004322 | ACAN | 4Y | M | no | yes |
| NP24FW2819 | HP:0001250 HP:0011355 HP:0000953 HP:0002832 | TSC2 | 4M | F | no | no |
| NP24FW2999 | HP:0001350 HP:0001288 | ABCD1 | 10Y | M | no | no |
| NP24FW3273 | HP:0001250 | SCN1A | 2Y | M | no | no |
| NP24FW3445 | HP:0002353 HP:0001263 HP:0012443 HP:0006705 HP:0001270 | MECP2 | 1Y | F | no | no |
| NP24FW3508 | HP:0001250 HP:0002151 HP:0000819 HP:0003074 | EIF2AK3 | 3M | M | no | no |
| NP24FW3777 | HP:0002719 HP:0001510 | CREBBP | 1Y | M | no | no |
| NP24FW4129 | HP:0001317 HP:0002353 HP:0001250 | TPP1 | 8Y | F | no | no |
| NP24FW4243 | HP:0000708 HP:0012758 | HECW2 | 1Y | F | no | no |
| NP24FW4300 | HP:0001250 HP:0000750 HP:0000729 HP:0002353 | GRIK2 | 5Y | M | no | no |
| NP24FW4293 | HP:0001217 HP:0100759 | HPGD | 2Y | M | no | no |
| NP24FW4296 | HP:0011927 HP:0004322 | ORC1 | 4Y | M | no | no |
| NP24FW4364 | HP:0012758 | EP300 | 2Y | M | no | no |
| NP24FW4349 | HP:0001252 HP:0000347 HP:0000765 HP:0000426 | MMACHC | 10D | M | no | no |
| NP24FW4393 | HP:0001249 HP:0001250 HP:0001252 | PIGN | 5Y | M | no | no |
| NP24FW4662 | HP:0001252 HP:0001873 HP:0000518 | OCRL | 1M | M | no | no |
| NP24FW4985 | HP:0004322 HP:0001508 | BRPF1 | 8Y | M | no | no |
| NP24FW5066 | HP:0001250 HP:0001622 HP:0025116 HP:0002014 | KCNQ2 | 13D | F | no | no |
| NP24FW5087 | HP:0001999 HP:0001510 HP:0000952 HP:0004322 HP:0004396 | OBSL1 | 4Y | M | no | no |
| NP24FW5151 | HP:0000953 HP:0001288 HP:0000849 HP:0002415 | ABCD1 | 9Y | M | no | no |

# Appendix II Causal-gene ranking trends for Exomiser along with minor factor adjustment

| Case ID | Exomiser V12.1 SUB.AF | Exomiser V12.1 default | Exomiser V12.1 FVF On | Exomiser V13.1 PROT.A | Exomiser V13.1 PROT.D |
| --- | --- | --- | --- | --- | --- |
| DDDP108234 | 12 | 12 | 4 | 3 | 2 |
| DDDP111242 | 1 | 1 | 1 | 1 | 1 |
| DDDP111137 | 13 | 12 | 6 | 3 | 1 |
| DDDP110985 | 58 | 49 | 19 | 3 | 1 |
| DDDP111681 | 20 | 18 | 7 | 1 | 1 |
| DDDP106414 | 11 | 10 | 2 | 2 | 1 |
| DDDP103148 | 32 | 28 | 18 | 11 | 27 |
| DDDP111001 | 6 | 6 | 2 | 2 | 1 |
| DDDP111456 | 15 | 14 | 8 | 4 | 1 |
| DDDP111390 | 3 | 2 | 1 | 1 | 1 |
| DDDP111496 | 14 | 13 | 5 | 3 | 1 |
| DDDP108406 | 27 | 24 | 6 | 1 | 1 |
| DDDP111423 | 15 | 13 | 3 | 4 | 1 |
| DDDP110970 | 17 | 16 | 9 | 3 | 1 |
| DDDP109893 | 2 | 2 | 1 | 1 | 2 |
| DDDP111406 | 17 | 14 | 8 | 4 | 2 |
| DDDP108233 | 5 | 5 | 1 | 1 | 1 |
| DDDP110961 | 324 | 271 | 114 | 4 | 3 |
| DDDP106875 | 79 | 72 | 27 | 8 | 1 |
| DDDP106064 | 11 | 9 | 5 | 3 | 9 |
| DDDP111116 | 3 | 3 | 2 | 1 | 17 |
| DDDP102138 | 14 | 14 | 6 | 6 | 2 |
| DDDP104582 | 31 | 26 | 11 | 3 | 1 |
| DDDP107622 | 14 | 12 | 7 | 4 | 1 |
| DDDP111262 | 5 | 4 | 3 | 3 | 1 |
| DDDP102251 | 15 | 13 | 3 | 2 | 1 |
| DDDP102213 | 2 | 1 | 1 | 1 | 1 |
| DDDP100213 | 3 | 2 | 1 | 1 | 1 |
| DDDP108828 | 8 | 7 | 2 | 2 | 2 |
| DDDP108825 | 6 | 6 | 2 | 2 | 1 |
| DDDP102680 | 5 | 3 | 1 | 1 | 1 |
| DDDP102589 | 1 | 1 | 1 | 1 | 1 |
| DDDP101834 | 141 | 118 | 43 | 20 | 6 |
| DDDP108896 | 8 | 8 | 3 | 2 | 1 |
| DDDP109017 | 3 | 3 | 3 | 3 | 12 |
| DDDP111698 | 165 | 137 | 41 | 27 | 10 |
| DDDP111151 | 1 | 1 | 1 | 1 | 1 |
| DDDP106760 | 2 | 2 | 1 | 1 | 1 |
| DDDP108473 | 9 | 8 | 1 | 1 | 1 |
| DDDP102284 | 27 | 20 | 11 | 38 | 9 |
| DDDP109995 | 352 | 298 | 67 | 48 | 12 |
| DDDP100243 | 60 | 55 | 19 | 6 | 1 |
| DDDP111317 | 153 | 132 | 60 | 37 | 6 |
| DDDP104617 | 10 | 10 | 4 | 3 | 2 |
| DDDP105700 | 1 | 1 | 1 | 1 | 1 |
| DDDP101230 | 32 | 27 | 10 | 4 | 1 |
| DDDP108148 | 4 | 3 | 1 | 1 | 1 |
| DDDP102392 | 2 | 2 | NA | NA | NA |
| DDDP110755 | 18 | 12 | 4 | 1 | 1 |
| DDDP109306 | 1 | 1 | 1 | 1 | 1 |
| DDDP110913 | 206 | 659 | 350 | 162 | 38 |
| DDDP102497 | 15 | 14 | 6 | 3 | 13 |
| DDDP102198 | 77 | 66 | 26 | 4 | 1 |
| DDDP100281 | 2 | 2 | 1 | 1 | 1 |
| DDDP102221 | 4 | 3 | 3 | 3 | 2 |
| DDDP112751 | 7 | 7 | 3 | 1 | 1 |
| DDDP110981 | 48 | 39 | 17 | 6 | 35 |
| DDDP109404 | 28 | 21 | 3 | 3 | 2 |
| DDDP101968 | 58 | 50 | 22 | 20 | 14 |
| DDDP110825 | 11 | 9 | 5 | 6 | 2 |
| DDDP110753 | 8 | 6 | 2 | 1 | 1 |
| DDDP110852 | 92 | 69 | 34 | 22 | 5 |
| DDDP111661 | 77 | 66 | NA | NA | NA |
| DDDP110879 | 30 | 28 | 14 | 13 | 3 |
| DDDP111266 | 5 | 5 | 1 | 1 | 1 |
| DDDP110890 | 61 | 57 | 28 | 18 | 2 |
| DDDP101989 | 1 | 1 | 1 | 1 | 1 |
| DDDP111313 | 19 | 18 | 7 | 4 | 1 |
| DDDP103619 | 52 | 42 | 19 | 12 | 8 |
| DDDP105749 | 10 | 7 | 6 | 2 | 1 |
| DDDP111271 | 3 | 3 | 1 | 1 | 1 |
| DDDP100121 | 13 | 12 | 7 | 7 | 4 |
| DDDP111486 | 3 | 3 | 3 | 3 | 1 |
| DDDP110962 | 36 | 35 | 10 | 4 | 2 |
| DDDP111469 | 2 | 2 | 2 | 3 | 1 |
| DDDP103664 | 1 | 1 | 1 | 1 | 1 |
| DDDP110920 | 7 | 7 | 5 | 1 | 1 |
| DDDP102057 | 1 | 1 | 1 | 1 | 1 |
| DDDP111129 | 1 | 1 | 1 | 1 | 1 |
| DDDP110855 | 3 | 3 | 2 | 2 | 1 |
| DDDP110133 | 39 | 30 | 16 | 8 | 28 |
| DDDP110801 | 10 | 10 | 3 | 2 | 1 |
| DDDP102594 | 16 | 14 | 4 | 4 | 1 |
| DDDP110164 | 5 | 4 | 3 | 2 | 1 |
| DDDP110916 | 4 | 4 | 2 | 1 | 1 |
| DDDP107502 | 17 | 15 | 5 | 3 | 1 |
| DDDP111330 | 20 | 18 | 6 | 5 | 1 |
| DDDP107459 | 2 | 2 | 2 | 2 | 1 |
| DDDP110896 | 7 | 6 | 2 | 2 | 1 |
| DDDP110826 | 557 | 699 | 247 | 126 | 28 |
| DDDP100298 | 224 | 190 | 66 | 44 | 8 |
| DDDP107416 | 49 | 41 | 16 | 8 | 3 |
| DDDP111178 | 8 | 7 | 3 | 2 | 1 |
| DDDP100284 | 8 | 8 | 5 | 3 | 1 |
| DDDP107056 | 2 | 1 | 1 | 1 | 1 |
| DDDP108556 | 6 | 6 | 4 | 3 | 2 |
| DDDP104933 | 23 | 20 | 6 | 3 | 3 |
| DDDP111463 | 66 | 56 | 29 | 37 | 9 |
| DDDP103343 | 41 | 22 | 9 | 5 | 1 |
| DDDP110960 | 3 | 2 | 1 | 2 | 1 |
| DDDP101224 | 30 | 27 | 12 | 8 | 2 |
| DDDP110794 | 15 | 12 | 8 | 3 | 1 |
| DDDP110889 | 85 | 69 | 15 | 13 | 3 |
| DDDP111043 | 2 | 2 | 1 | 1 | 2 |
| DDDP100320 | 27 | 19 | 10 | 7 | 1 |
| DDDP111759 | 12 | 8 | 5 | 2 | 1 |
| DDDP109511 | 33 | 25 | 15 | 5 | 2 |
| DDDP111065 | 2 | 2 | 1 | 1 | 1 |
| DDDP105431 | 338 | 284 | 105 | 81 | 9 |
| DDDP103532 | 2 | 2 | 2 | 3 | 2 |
| DDDP100283 | 6 | 5 | 3 | 2 | 1 |
| DDDP110720 | 115 | 88 | 29 | 24 | 4 |
| DDDP104625 | 216 | 175 | 71 | 9 | 2 |
| DDDP111298 | 21 | 18 | 3 | 4 | 2 |
| DDDP108103 | 3 | 3 | 2 | 2 | 1 |
| DDDP110777 | 11 | 11 | 1 | 1 | 1 |
| DDDP107324 | 3 | 3 | 2 | 2 | 1 |
| DDDP111682 | 136 | 109 | 33 | 18 | 4 |
| DDDP106128 | 77 | 65 | 26 | 10 | 2 |
| DDDP101699 | 155 | 77 | 31 | 16 | 6 |
| DDDP102140 | 11 | 9 | 5 | 3 | 1 |
| DDDP111060 | 16 | 18 | 10 | NA | NA |
| DDDP100224 | 5 | 4 | 2 | 1 | 1 |
| DDDP102111 | 46 | 38 | 10 | 6 | 2 |
| DDDP111249 | 1 | 1 | 1 | 1 | 1 |
| DDDP104052 | 1 | 1 | 1 | 1 | 2 |
| DDDP100184 | 132 | 109 | 23 | 29 | 7 |
| DDDP111146 | 1 | 1 | 1 | 1 | 4 |
| DDDP105942 | 2 | 2 | 1 | 3 | 1 |
| DDDP104519 | 34 | 33 | 11 | 3 | 2 |
| DDDP111595 | 33 | 25 | 10 | 6 | 3 |
| DDDP100161 | 42 | 37 | 11 | 5 | 20 |
| DDDP110983 | 1 | 1 | 1 | 1 | 1 |
| DDDP105140 | 19 | 17 | 6 | 3 | 1 |
| DDDP111105 | 8 | 5 | 3 | 3 | 1 |
| DDDP101851 | 86 | 65 | 16 | 3 | 1 |
| DDDP110121 | 5 | 4 | 3 | 3 | 1 |
| DDDP101866 | 1 | 1 | 1 | 1 | 1 |
| DDDP111361 | 17 | 14 | 7 | 6 | 1 |
| DDDP102578 | 30 | 25 | 4 | 3 | 3 |
| DDDP111254 | 2 | 2 | 1 | 1 | 1 |
| DDDP110872 | 161 | 132 | 37 | 15 | 6 |
| DDDP110824 | 16 | 15 | 5 | 4 | 1 |
| DDDP110717 | 23 | 17 | 4 | 1 | 1 |
| DDDP111211 | 3 | 2 | 1 | 1 | 1 |
| DDDP111619 | 1 | 1 | 1 | 1 | 1 |
| DDDP104896 | 4 | 2 | 1 | 2 | 1 |
| DDDP112647 | 6 | 5 | 1 | 2 | 26 |
| DDDP102189 | 4 | 4 | 3 | 2 | 1 |
| DDDP111580 | 103 | 92 | 27 | 6 | 2 |
| DDDP111292 | 10 | 10 | 6 | 5 | 3 |
| DDDP110892 | 1 | 1 | 1 | 1 | 1 |
| DDDP110127 | 3 | 3 | NA | NA | NA |
| DDDP111281 | 27 | 21 | 9 | 7 | 2 |
| DDDP109883 | 37 | 33 | 11 | 5 | 2 |
| DDDP111081 | 1 | 1 | 1 | 1 | 1 |
| DDDP103107 | 1 | 1 | 1 | 1 | 25 |
| DDDP102205 | 24 | 22 | NA | NA | NA |
| DDDP103044 | 18 | 16 | 5 | 2 | 1 |
| DDDP111175 | 1 | 1 | 1 | 1 | 1 |
| DDDP110738 | 1 | 1 | 1 | 1 | 1 |
| DDDP111288 | 18 | 16 | 8 | 4 | 1 |
| DDDP111459 | 19 | 15 | 4 | 3 | 1 |
| DDDP105767 | 12 | 11 | 3 | 1 | 1 |
| DDDP111219 | 13 | 8 | 2 | 1 | 1 |
| DDDP103710 | 11 | 8 | 2 | 1 | 1 |
| DDDP102894 | 87 | 67 | 17 | 12 | 5 |
| DDDP110761 | 40 | 34 | 11 | 6 | 1 |
| DDDP111170 | 3 | 3 | 1 | 1 | 1 |
| DDDP108836 | 6 | 5 | 2 | 2 | 1 |
| DDDP106936 | 29 | 24 | 5 | 3 | 1 |
| DDDP100187 | 17 | 14 | 4 | 2 | 1 |
| DDDP104755 | 3 | 2 | 1 | 1 | 1 |
| DDDP103666 | 7 | 5 | 2 | 1 | 1 |
| DDDP111799 | 21 | 18 | 9 | 7 | 9 |
| DDDP112690 | 19 | 16 | 7 | 5 | 2 |
| DDDP107978 | 5 | 5 | 3 | 2 | 1 |
| DDDP111141 | 12 | 12 | 7 | 1 | 1 |
| DDDP111487 | 69 | 52 | 20 | 6 | 1 |
| DDDP111246 | 8 | 7 | 4 | 3 | 1 |
| DDDP110776 | 38 | 33 | 10 | 5 | 1 |
| DDDP105825 | 1 | 1 | 1 | 1 | 1 |
| DDDP100030 | 16 | 12 | 4 | 4 | 3 |
| DDDP105741 | 27 | 20 | 2 | 4 | 1 |
| DDDP111238 | 108 | 94 | 36 | 17 | 4 |
| DDDP110748 | 1 | 1 | 1 | 1 | 1 |
| DDDP111286 | 6 | 5 | 2 | 2 | 2 |
| DDDP100175 | 2 | 2 | 1 | 6 | 2 |
| DDDP103059 | 42 | 36 | 13 | 8 | 5 |
| DDDP110713 | 8 | 5 | 2 | 3 | 1 |
| DDDP111138 | 111 | 94 | 34 | 106 | 17 |
| DDDP110982 | 34 | 31 | 14 | 11 | 17 |
| DDDP110976 | 1 | 1 | 1 | 1 | 1 |
| DDDP111027 | 1 | 1 | 1 | 1 | 1 |
| DDDP110736 | 4 | 2 | 2 | 1 | 1 |
| DDDP111433 | 8 | 7 | 5 | 3 | 1 |
| DDDP111265 | 77 | 66 | 12 | 6 | 2 |
| DDDP112654 | 12 | 10 | NA | NA | NA |
| DDDP111400 | 20 | 15 | 9 | 5 | 2 |
| DDDP111106 | 74 | 68 | 37 | 16 | 4 |
| DDDP102117 | 1 | 1 | 1 | 1 | 1 |
| DDDP105217 | 2 | 2 | 2 | 1 | 1 |
| DDDP111133 | 38 | 29 | 10 | 4 | 1 |
| DDDP110722 | 12 | 11 | 4 | 2 | 1 |
| DDDP111128 | 94 | 41 | 17 | 8 | 2 |
| DDDP101115 | 34 | 33 | 10 | 6 | 20 |
| DDDP111119 | 10 | 8 | 5 | 4 | 1 |
| DDDP106343 | 19 | 16 | 9 | 6 | 12 |
| DDDP103467 | 5 | 5 | 3 | 4 | 1 |
| DDDP111460 | 7 | 5 | 2 | 3 | 156 |
| DDDP111417 | 1 | 1 | 1 | 1 | 1 |
| DDDP111627 | 9 | 6 | 2 | 2 | 2 |
| DDDP109873 | 37 | 31 | 7 | 3 | 1 |
| DDDP111503 | 32 | 27 | 12 | 10 | 2 |
| DDDP111123 | 69 | 56 | 20 | 13 | 4 |
| DDDP110659 | 8 | 6 | 2 | 1 | 1 |
| DDDP100091 | 4 | 3 | 2 | 3 | 1 |
| DDDP102547 | 4 | 3 | 1 | 9 | 2 |
| DDDP105476 | 3 | 3 | 1 | 1 | 1 |
| DDDP101852 | 16 | 16 | 3 | 2 | 2 |
| DDDP110766 | 93 | 77 | 28 | 10 | 15 |
| DDDP102168 | 36 | 33 | 15 | 11 | 1 |
| DDDPNA9 | 1 | 1 | 1 | 1 | 1 |
| DDDP107364 | 6 | 4 | 2 | 1 | 1 |
| DDDP111181 | 136 | 113 | 32 | 21 | 1 |
| DDDP111217 | 13 | 11 | 5 | 4 | 1 |
| DDDP102759 | 5 | 4 | 2 | 2 | 1 |
| DDDP111190 | 20 | 17 | 6 | 4 | 1 |
| DDDP111703 | 18 | 15 | 5 | 4 | 1 |
| DDDP110856 | 1 | 1 | 1 | 1 | 1 |
| DDDP102114 | 48 | 44 | 19 | 6 | 1 |
| DDDP112775 | 26 | 23 | 7 | 6 | 3 |
| DDDP111391 | 16 | 15 | 7 | 5 | 2 |
| DDDP111198 | 1 | 1 | 1 | 1 | 1 |
| DDDP103122 | 27 | 23 | 8 | 19 | 3 |
| DDDP108198 | 38 | 33 | 9 | 6 | 1 |
| DDDP108492 | 7 | 5 | 1 | 1 | 1 |
| DDDP110796 | 1 | 1 | 1 | 1 | 1 |
| DDDP100287 | 11 | 10 | 4 | 3 | 3 |
| DDDP107419 | 30 | 27 | 11 | 5 | 1 |
| DDDP107421 | 72 | 60 | 19 | 13 | 4 |
| DDDP106981 | 29 | 23 | 8 | 3 | 1 |
| DDDP102411 | 2 | 1 | 1 | 1 | 1 |
| DDDP105451 | 3 | 3 | 2 | 1 | 1 |
| DDDP101836 | 5 | 4 | 1 | 2 | 1 |
| DDDP104121 | 1 | 1 | 1 | 1 | 1 |
| DDDP104795 | 2 | 2 | 1 | 2 | 4 |
| DDDP110818 | 31 | 29 | 12 | 8 | 4 |
| DDDP105423 | 13 | 8 | 4 | 4 | 17 |
| DDDP110662 | 42 | 34 | 15 | 9 | 3 |
| DDDP104532 | 15 | 11 | 7 | 7 | 2 |
| DDDP101995 | 5 | 4 | 3 | 2 | 1 |
| DDDP108105 | 2 | 2 | 1 | 1 | 1 |
| DDDP111187 | 2 | 2 | 1 | 1 | 1 |
| DDDP111218 | 1 | 1 | 1 | 1 | 1 |
| DDDP111253 | 9 | 7 | 1 | 1 | 1 |
| DDDP102206 | 6 | 6 | 3 | 2 | 1 |
| DDDP111465 | 11 | 6 | 4 | 3 | 2 |
| DDDP108015 | 49 | 45 | 19 | 7 | 1 |
| DDDP111596 | 2 | 1 | 1 | 1 | 1 |
| DDDP103048 | 1 | 1 | 1 | 1 | 1 |
| DDDP111775 | 52 | 45 | 13 | 3 | 1 |
| DDDP102389 | 1 | 1 | 1 | 1 | 1 |
| DDDP111250 | 1 | 1 | 1 | 1 | 1 |
| DDDP101867 | 29 | 25 | 3 | 2 | 1 |
| DDDP104968 | 10 | 6 | 3 | 3 | 2 |
| DDDP111392 | 11 | 8 | 2 | 2 | 1 |
| DDDP111221 | 1 | 1 | 1 | 1 | 1 |
| DDDP106791 | 1 | 1 | 1 | 1 | 1 |
| DDDP111468 | 2 | 2 | 2 | 1 | 2 |
| DDDP111239 | 7 | 6 | 2 | 2 | 1 |
| DDDP107971 | 3 | 3 | 3 | 2 | 1 |
| DDDP111227 | 107 | 93 | 29 | 18 | 4 |
| DDDP108745 | 1 | 1 | 1 | 1 | 1 |
| DDDP102294 | 18 | 17 | 7 | 4 | 1 |
| DDDP105223 | 16 | 14 | 4 | 3 | 1 |
| DDDP111515 | 3 | 3 | 2 | 2 | 1 |
| DDDP106372 | 32 | 26 | 12 | 6 | 4 |
| DDDP100160 | 3 | 2 | 1 | 1 | 1 |
| DDDP105394 | 7 | 7 | 2 | 3 | 1 |
| DDDP111350 | 34 | 29 | 8 | 19 | 3 |
| DDDP110784 | 1 | 1 | 1 | 1 | 1 |
| DDDP110760 | 1 | 1 | 1 | 1 | 1 |
| DDDP110929 | 7 | 6 | 5 | 3 | 3 |
| DDDP112720 | 9 | 7 | NA | NA | NA |
| DDDP102761 | 127 | 105 | 27 | 13 | 3 |
| DDDP111285 | 1 | 1 | 1 | 1 | 1 |
| DDDP108415 | 7 | 7 | 4 | 2 | 1 |
| DDDP107008 | 15 | 14 | 5 | 4 | 2 |
| DDDP111096 | 1 | 1 | 1 | 2 | 1 |
| DDDP111104 | 8 | 7 | 1 | 1 | 1 |
| DDDP105999 | 6 | 5 | 2 | 2 | 1 |
| DDDP111224 | 488 | 411 | 155 | 85 | 10 |
| DDDP100120 | 91 | 72 | 26 | 14 | 2 |
| DDDP108067 | 5 | 5 | 3 | 2 | 1 |
| DDDP103071 | 4 | 3 | 3 | 1 | 1 |
| DDDP102820 | 28 | 26 | 10 | 5 | 2 |
| DDDP111214 | 5 | 4 | 2 | 2 | 2 |
| DDDP111204 | 68 | 55 | 24 | 15 | 1 |
| DDDP111154 | 8 | 6 | 1 | 1 | 1 |
| DDDP111333 | 11 | 11 | 2 | 1 | 1 |
| DDDP107471 | 12 | 10 | 4 | 2 | 3 |
| DDDP105270 | 10 | 9 | 2 | 1 | 1 |
| DDDP106882 | 14 | 12 | 3 | 1 | 1 |
| DDDP102726 | 3 | 2 | 1 | 1 | 1 |
